# Evolvability-enhancing mutations in the fitness landscapes of an RNA and a protein

**DOI:** 10.1038/s41467-023-39321-8

**Published:** 2023-06-19

**Authors:** Andreas Wagner

**Affiliations:** 1 Department of Evolutionary Biology and Environmental Studies, University of Zurich, Zurich, Switzerland; 2 Swiss Institute of Bioinformatics, Quartier Sorge-Batiment Genopode, Lausanne, Switzerland; 3 The Santa Fe Institute, Santa Fe, NM USA

**Keywords:** Molecular evolution, Evolutionary genetics, Evolutionary theory

## Abstract

Can evolvability—the ability to produce adaptive heritable variation—itself evolve through adaptive Darwinian evolution? If so, then Darwinian evolution may help create the conditions that enable Darwinian evolution. Here I propose a framework that is suitable to address this question with available experimental data on adaptive landscapes. I introduce the notion of an evolvability-enhancing mutation, which increases the likelihood that subsequent mutations in an evolving organism, protein, or RNA molecule are adaptive. I search for such mutations in the experimentally characterized and combinatorially complete fitness landscapes of a protein and an RNA molecule. I find that such evolvability-enhancing mutations indeed exist. They constitute a small fraction of all mutations, which shift the distribution of fitness effects of subsequent mutations towards less deleterious mutations, and increase the incidence of beneficial mutations. Evolving populations which experience such mutations can evolve significantly higher fitness. The study of evolvability-enhancing mutations opens many avenues of investigation into the evolution of evolvability.

## Abstract (web summary)

Whether evolvability itself can be a product of adaptive Darwinian evolution is a debated question. This study proposes that adaptive landscapes harbor mutations that enhance the evolvability of evolving molecules and help populations of these molecules to evolve high fitness.

## Introduction

All biological systems are to some extent evolvable. That is, they are capable of bringing forth variation that is both adaptive and heritable, and that enables them to respond to natural selection. The causes of such evolvability are increasingly well-studied^1–6^. They are also very broad. At one extreme are global organizational features of living systems, such as the rate at which they create DNA mutations, or their ability to buffer the deleterious effects of mutations. Some of these features, such as the structure of the genetic code, have been in place since life’s early days, and they no longer change substantially^7^. At the other extreme are more ephemeral properties^8–11^, such as the location of evolving organisms in a fitness landscape—an analog of a physical landscape that helps to understand the dynamics of Darwinian evolution^12^. Such local properties can change very rapidly in evolution. For example, single-point mutations can alter the evolvability of bacterial populations, and predispose them to adapt more rapidly to a given environment^8–10^.

Much less well-studied than the causes of evolvability is their evolution. Can evolvability itself be a product of adaptive Darwinian evolution?^4, 5^ The question is important, because a positive answer implies that Darwinian evolution can help create the conditions under which Darwinian evolution becomes possible. Here I address part of this question from a genetic perspective. That is, I identify and study DNA mutations that increase evolvability. And I define such mutations by how they influence the phenotypic effects of subsequent mutations.

A major challenge in answering the above question is that evolvability is a dispositional trait. It refers to the potential of a biological system to bring forth adaptive variation, for example through DNA mutations^13–15^. As opposed to traits that directly affect an organism’s survival or reproduction, and are thus directly affected by natural selection, dispositional traits may be subject to a more indirect form of selection on their future consequences. Such selection has also been called second-order selection, to distinguish it from direct (first-order) selection. It can enhance evolvability only under restrictive conditions, such as large population sizes or high mutation rates, which are not met by all organisms^16–19^.

This problem can in principle be overcome through DNA mutations that increase not only the evolvability of an organism but also its fitness^20^. Such DNA mutation would be able to spread through a population via their direct effects on fitness, without requiring second-order selection. And in doing so, they could also enhance evolvability. Such “dual effect” mutations—evolvability-enhancing and fitness-enhancing—do indeed exist^21–25^. For example, a recent laboratory evolution experiment aiming to change the color of a fluorescent protein’s light emission identified mutations that change both the fitness (fluorescence intensity) and the evolvability of the protein. They did so by increasing the ability of the protein to fold, which increased its potential to accommodate mutations that can increase fitness even further^25^.

Because evolution experiments are not designed to quantify the incidence of such mutations, I here use different kinds of data to identify and characterize evolvability-enhancing mutations. This data comes from experimentally characterized fitness landscapes^26,27^. Each location in such a landscape corresponds to a genotype. The elevation at that location is the fitness of that genotype. Current technologies allow us to measure the fitness or a proxy of fitness for thousands of protein or RNA genotypes within a larger collection or “space” of such genotypes.^26,28–43^, and to analyze the topography of the resulting landscape. A small subset of landscapes characterized to date are combinatorially complete or nearly so^26,27,34,43–46^. In a combinatorially complete landscape, for any two genotypes whose fitness is known, the fitness of all genotypes that lie on the shortest mutational paths between these genotypes is also known. Combinatorial completeness is important for my purpose, because it permits the evaluation of a mutant genotype and its interactions with other genotypes in multiple genetic backgrounds.

I will first define the notion of an evolvability-enhancing (EE) mutation quantitatively. I will focus mostly on beneficial EE mutations, which themselves increase fitness while at the same time enhancing evolvability, because beneficial mutations spread most easily through populations via direct selection. I then study the incidence and distribution of fitness effects of EE mutations, as well as their evolutionary dynamics in experimentally characterized adaptive landscapes. The landscapes I study must fulfill several criteria. First, organismal fitness and not just a proxy of fitness must have been measured in vivo, in the form of a microbial growth rate. Second, the landscapes need to be large (>10^3^ genotypes), partly because EE mutations may be rare, and it may thus be necessary to evaluate many mutations for their effects on fitness and evolvability. Finally, the landscapes need to be combinatorially complete or nearly so. These requirements are fulfilled by few experimentally characterized landscapes^26,27,46^. I chose two that represent different kinds of molecules and organisms. The first is the protein fitness landscape of an *E. coli* toxin-antitoxin system, which comprises fitness values for 7882 antitoxin protein genotypes^27^. The second is an RNA fitness landscape of 4176 yeast (*Saccharomyces cerevisiae*) transfer RNA (tRNA) genotypes.

In this work, I use these landscapes to show that EE mutations exist and comprise a small minority of all mutations that can improve the fitness effects of subsequent mutations dramatically. A population that encounters evolvability-enhancing mutations during adaptive evolution on such landscapes can achieve significantly higher fitness.

## Results

### Defining evolvability-enhancing (EE) mutations

To define an evolvability-enhancing (EE) DNA mutation, consider some wild-type genotype ${wt}$ with fitness $w({wt})$ and a 1-mutant neighbor $m$ with fitness $w\left(m\right),$ i.e., a genotype that can be produced from the wild-type through a point mutation that changes a single nucleotide in the wild-type. Even though the word enhancing may suggest that an evolvability-enhancing mutation actively changes the effects of other mutations, that would be an unintended anthropomorphism. An EE mutation simply creates a genetic background $m$ in which mutations are more likely to be adaptive than in the wild-type background ${wt}$.

To make this property more precise, I first denote the set of all 1-mutant neighbors of the ${wt}$ (excluding $m$ itself) as $\left\{{n}_{{wt}}\right\}=$ $\left\{{n}_{{wt},1},\ldots,{n}_{{wt},k}\right\}.$ In other words, these are all genotypes that can be reached through a single mutation from the wild-type genetic “background”. Denote their fitness values as $\left\{{w}({n}_{{wt},1}),\ldots,{w}({n}_{{wt},k})\right\}$ and their mean fitness as $\bar{w}({n}_{{wt}})$. Likewise, denote the set of all 1-mutant neighbors of $m$ (excluding the ${wt}$) as $\left\{{n}_{m}\right\}=$ $\left\{{n}_{m,1},\ldots,{n}_{m,k}\right\}$. All these are genotypes that can be reached through a single mutation in the mutant genetic background. Denote their fitness values as $\left\{{w}({n}_{m,1}),\ldots,{w}({n}_{m,k})\right\}$ and their mean fitness as $\bar{w}({n}_{m})$.

**Fig. 1:**
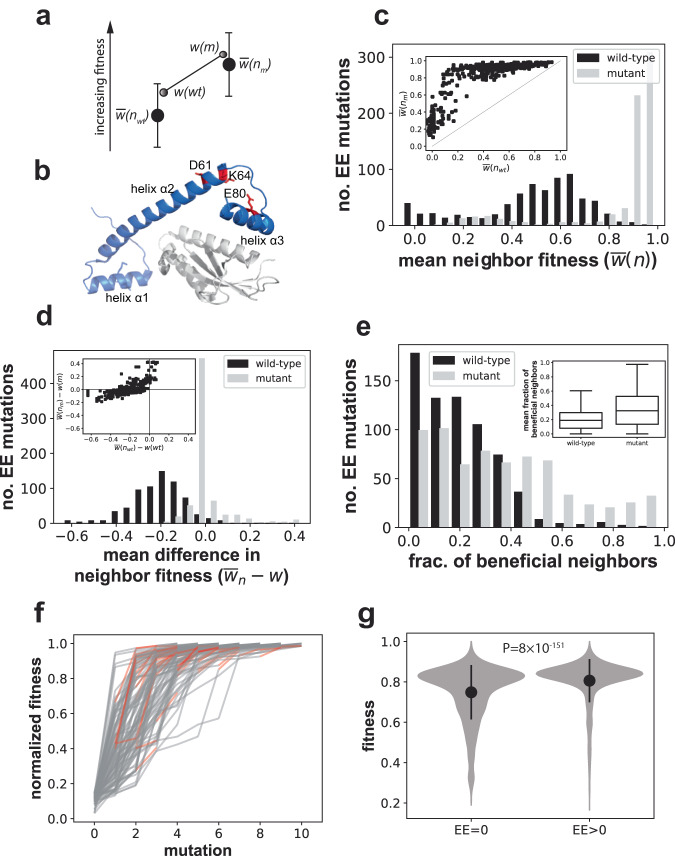
Beneficial EE mutations in a protein adaptive landscape. **a** The notion of a beneficial EE mutation. Gray circles indicate the fitness $\bar{w}$ of a hypothetical wild-type (*wt*) and mutant (*m*) genotype. Lower (upper) black circles and vertical bars indicate mean and standard deviation of the fitness of the 1-mutant neighbors of the hypothetical wild-type (mutant). Each black circle is drawn below the nearest gray circle to reflect the observation that mutations are on average deleterious^50^. An EE mutation reduces the deleterious effects of mutations and increases the incidence of beneficial mutations, as indicated by the shorter distance of the upper black circle to the gray circle symbolizing mutant fitness**. b** Tertiary structure of the ParD3 antitoxin (blue) in complex with its cognate toxin ParE3 (gray; protein database (PDB) file: 5CEG; 10.2210/pdb5CEG/pdb; ref. ^74^). The residues D61, K64, and E80 (red) of the *M. opportunistum* reference antitoxin sequence are part of antitoxin helix 2 and 3^27^**. c** Distribution of the mean fitness of all neighbors of the wild-type ($\bar{w}\left({n}_{{wt}}\right),$ black), as well as of all neighbors of the mutant ($\bar{w}\left({n}_{m}\right),$ gray), for all pairs of neighbors and their beneficial EE mutants in the ParD3 antitoxin fitness landscape of^27^. Inset: scatterplot of $\bar{w}\left({n}_{{wt}}\right)$ and $\bar{w}\left({n}_{m}\right)$; diagonal: $\bar{w}\left({n}_{{wt}}\right)=\bar{w}\left({n}_{m}\right)$. **d** Distribution of the mean fitness of all neighbors of the wild-type adjusted by the fitness of the wild-type ($\bar{w}\left({n}_{{wt}}\right)-w({wt}),$ black), as well as of all neighbors of the mutant adjusted by the fitness of the mutant ($\bar{w}\left({n}_{m}\right)-w(m),$ gray), for all pairs of neighbors and their beneficial EE mutants in the protein landscape. Inset: scatterplot of the same quantities. **e** Distribution of the fraction of beneficial neighbors (neighbors with greater fitness) of the wild-type (black) as well as of the corresponding mutant (gray), for all *n* = 681 pairs of wild-type sequences and the corresponding beneficial EE mutants. Inset: box plot of this fraction (box height: interquartile range (IQR), horizontal bar: median; whisker length: 1.5 × IQR) **f** fitness evolution (vertical axis) during 100 stochastic adaptive walks on the protein landscape. Red edges correspond to EE mutations that occur during an adaptive walk**. g** The fitness difference attained after step three of 10^4^ adaptive walks between walkers that experienced no (EE = 0) and at least one (EE > 0) EE mutations is highly significant (*P* = 8 × 10^−151^, two-sided Mann–Whitney *U* = 7,390,352, *n* = 6778 independent random walks). Circles and vertical bars show means and one standard deviation. Fitness values in (**f**, **g**) are normalized to lie in the interval (0,1). Source data are provided as a Source Data file.

One possible definition of an EE mutation is that $m$ must increase the fitness of all possible such point mutations. In other words, one might require that the fitness of each mutant in $\left\{{n}_{m}\right\}$ should be higher than the fitness of each mutant in $\left\{{n}_{{wt}}\right\}$: ${w}({n}_{m,i})\, > \,{w}({n}_{{wt},i})$ for all neighbors $i$. However, this would be an unrealistic expectation, because some mutations (such as nonsense mutations) may always be equally deleterious, regardless of the genetic background in which they occur. For this reason, I will only require that an EE mutation increases the fitness benefit of subsequent mutations on average (Fig. 1a).

The following considerations apply to both beneficial and neutral EE mutations ($\Delta w=w\left(m\right)-w({wt})\ge 0$; see Supplementary Methods for a definition of deleterious EE mutations).

For a neutral mutation $(\Delta w=0)$ to be EE, I require that

1 $$\bar{w}\left({n}_{m}\right)-\bar{w}({n}_{{wt}})\, > \,0$$

This means that the mutation $m$ causes the fitness of mutations that occur in its background to be higher than that of mutations in the wild-type background on average. If $m$ is beneficial, this condition needs to be modified for the following reason. If mutations interact additively in their effect on fitness, the condition $\bar{w}\left({n}_{m}\right)-\bar{w}({n}_{{wt}})$ > 0 would be trivially met as a result of the fact that the mutation $m$ itself is beneficial $\left(\Delta w > 0\right)$. To see this, note that in case of additivity

$$\bar{w}({n}_{m})-\bar{w}({n}_{{wt}})	=\left(\frac{1}{k}\right)\mathop{\sum }\limits_{i=1}^{k}({w}({n}_{m,i})-{w}({n}_{{wt},i})) \\ 	=\left(\frac{1}{k}\right)\mathop{\sum }\limits_{i=1}^{k}({w}({n}_{{wt},i})+\Delta w-{w}({n}_{{wt},i}))=\Delta w > 0$$

Thus, for a beneficial mutation *m* to be EE, it is necessary to require more, namely that *m* increases the fitness of other mutations beyond its own fitness benefit, that is:

2 $$\bar{w}\left({n}_{m}\right)-\bar{w}({n}_{{wt}})\, > \,\Delta w$$

This requirement is mathematically equivalent to

3 $$\bar{w}\left({n}_{m}\right)-w\left(m\right)\, > \,\bar{w}\left({n}_{{wt}}\right)-w\left({wt}\right)$$

In plain words, the neighbors of *m* must have higher fitness (relative to $m$) than the neighbors of the wild-type (relative to the wild-type). This condition is equivalent to requiring that $m$ shows on average positive epistasis with the mutations leading to its neighbors (Supplementary Methods). I define but do not explicitly study neutral EE mutations further here, because such mutations cannot be reliably identified in large populations with today’s technology to measure fitness.

### EE mutations are rare in the protein adaptive landscape

I first study the fitness landscape formed by genetic variants at three amino acid positions of the antitoxin protein ParD3 from the pseudomonad bacterium *Mesorhizobium opportunistum* (Fig. 1b and Supplementary Methods). When expressed in *E. coli*, this protein is one member of a toxin-antitoxin pair whose specific binding to the toxin protein ParE3 is necessary for *E. coli* growth. Mutations in the antitoxin that reduce such binding allow the toxin to inhibit cell growth through its interaction with DNA gyrase^47^. Toxin-antitoxin pairs like these are widespread in bacteria, occur both on plasmids and chromosomes, are frequently horizontally transferred, and may have important biological roles, such as to help maintain plasmids in a population^48, 49^.

I use data from a study whose authors employed mutagenesis to randomize three key antitoxin amino acids that affect the antitoxin’s binding specificity to the toxin and thus fitness^27^ (Fig. 1b). I will refer here to the genotype of the unmutated antitoxin (D61, K64, and E80) as the reference or *M. opportunistum* genotype. Starting from this reference, the authors created a library of all possible 20^3^ = 8000 antitoxin variants, expressed them from plasmids in *E. coli*, and measured the fitness of *E. coli* cells expressing these variants through Illumina sequencing after mass selection^27^ (Supplementary Methods). The published data contains fitness information on 98.5% (7882) of these variants. I analyze this data as an adaptive landscape, and consider those variants neighbors of each other that differ in a single amino acid, and where the differing amino acids can be reached from each other through a single-point mutation (“Methods”).

I first examined all 175,552 pairs of neighboring variants, considered one member of the pair as the “wild-type”, and asked whether the mutation creating the neighbor meets criterion (2) for being beneficial and evolvability-enhancing (“Methods”). I found that such beneficial EE mutations comprise only 0.39% (681) of all mutations in the landscape. Figure 1c shows histograms of the mean fitness of the neighbors of the wild-type (black) as well as of the mutant (gray), for all beneficial EE mutations. The gray histogram is shifted far to the right, indicating that the mean fitness of the neighbors of the mutant is substantially higher than that of the neighbors of the wild-type. Indeed, when averaged over all beneficial EE mutations, the mean fitness of the neighbors of the mutant $\bar{w}\left({n}_{m}\right)=$ 0.85, which is 77% higher than the mean fitness of the wild-type $\bar{w}\left({n}_{{wt}}\right)=$ 0.48, a difference that is highly significant (*P* = 3.8 × 10^−148^, two-sided Mann–Whitney *U* = 43,750, *n* = 681).

Considering only the difference in the fitness of neighbors of a mutant and of a wild-type ignores the fitness differences that exist between the wild-type and the mutant themselves. To take this fitness difference into account, Fig. 1d shows histograms of the mean fitness of the wild-type’s neighbors adjusted for the fitness of the wild-type ($\bar{w}\left({n}_{{wt}}\right)-w\left({wt}\right)$), as well as the analogous quantity for the mutants ($\bar{w}\left({n}_{m}\right)-w\left(m\right)$). Even with this adjustment, the pronounced right shift of the distribution persists. More specifically, averaged over all beneficial EE mutations $\bar{w}\left({n}_{{wt}}\right)-w\left({wt}\right)$ = −0.21 and $\bar{w}\left({n}_{m}\right)-w\left(m\right)$ = −0.004, a difference that is again highly significant (*P* = 4.9 × 10^−185^, two-sided Mann–Whitney *U* = 21329, *n* = 681). I note that the sign of both quantities is negative, which is consistent with the general principle that mutations are on average deleterious^50^. This observation raises the question whether EE mutations, as defined here, only render deleterious mutations more weakly deleterious on average, instead of increasing the propensity of mutations to be beneficial? The inset of Fig. 1d, which plots $\bar{w}\left({n}_{m}\right)-w\left(m\right)$ against $\bar{w}\left({n}_{{wt}}\right)-w\left({wt}\right)$, already hints that this is not the case. Its upper left quadrant contains EE mutations whose neighbors are on average beneficial ($\bar{w}\left({n}_{m}\right)-w\left(m\right)$ > 0), whereas the same mutations in the wild-type are on average deleterious ($\bar{w}\left({n}_{{wt}}\right)-w\left({wt}\right)$ < 0). There are 119 such mutations or 17.4% of all beneficial EE mutations. Figure 1e shows the distribution of the fraction of beneficial neighbors both for the wild-type and each corresponding EE mutant. This distribution is again shifted rightward, towards a higher fraction of beneficial mutation in the neighborhood of EE mutations. Overall, 20.6% of wild-type neighbors are on average beneficial, whereas 36.3% of EE mutant neighbors are (Fig. 1e, inset), an increase of 76% that is statistically highly significant (*P* = 4.9 × 10^−32^, two-sided Mann–Whitney *U* = 146,432, *n* = 681). In addition, 72.5% of EE mutants (494 of 681) have more beneficial neighbors than their wild-type ancestors. In sum, EE mutations do not just shift the distribution of mutational effect toward less deleterious mutations. Their neighbors are also more likely to be beneficial. Deleterious EE mutations share these main properties with beneficial EE mutations (Supplementary Fig. 1).

Beneficial EE mutations are rare in the lowest fitness regions of the landscape (Supplementary Fig. 3a). However, beneficial EE mutations do not themselves cause higher or lower fitness gains $\left(\Delta w\right)$ than other beneficial mutations (*P* = 0.34, two-sided Mann–Whitney *U* = 29,022,948, *n*_*1*_ = 681, *n*_*2*_ = 87076; mean $\Delta w$=0.16 and mean $\Delta w$=0.17 for beneficial EE mutations and beneficial non-EE mutations).

An examination of the most strongly evolvability-enhancing mutations shows that they fall into only three categories. They comprise mutations towards the amino acid D at position 61 (Fig. 1b), mutations toward the amino acid K at position 64, and mutations away from a proline at position 81 (Supplementary Table 1). All of these changes show pronounced nonadditive interactions with subsequent mutations that render such mutations less deleterious and more likely to be beneficial (Supplementary Table 1).

### Adaptive walks with EE mutations lead to higher fitness in the protein landscape

I next asked whether the occurrence of an evolvability-enhancing mutation in an evolving population would influence the speed of adaptive evolution. To model adaptive evolution, I mostly consider a weak-mutation scenario^18,51,52^, because I study the evolution of a small section of a gene in which mutations are much rarer than in whole genomes. In this scenario, adaptive evolution can be modeled as an adaptive random walk of a single genotype changing through point mutations (“Methods”).

I modeled this adaptive walk stochastically, using Kimura’s formula for the probability ${p}_{{fix}}$ that a mutation goes to fixation in a haploid population under the influence of selection and drift (“Methods”). At the large population sizes of *E. coli* (*N* = 1.8 × 10^8^)^53^, deleterious mutations are very unlikely to go to fixation, such that beneficial mutations dominate the evolutionary dynamics.

I analyzed 10^4^ stochastic adaptive walks of maximally ten mutational steps each, which started out from randomly chosen genotypes from the bottom 5% of the fitness distribution, and for a population size similar to that of *E. coli* (*N* = 10^8^)^53^. An adaptive walk can terminate prematurely if it reaches a global or local fitness peak, i.e., a genotype whose neighbors all have fitness lower than itself. Figure 1f shows the evolutionary trajectories of a sample of 100 of these walks, with EE-enhancing mutation shown in red. The figure shows that adaptive walks approach the highest possible fitness values within a mere few steps, implying that the landscape is highly navigable. In fact, by the fourth step, the median fitness among all 10^4^ walks has reached more than 90% of the maximally possible fitness (median $w=0.94$). Thus, EE mutations can make a difference only early during a adaptive walk, before most trajectories have reached high fitness. Here I study the preceding (third) step of the adaptive walks, at which the difference between the maximum and the minimum fitness among all walks is 0.8, i.e., it covers 80% of the possible fitness range. I determined the average fitness increase that the 10^4^ adaptive walks had achieved by the third step of the adaptive walk. This increase was 7.7% higher for adaptive walks that had experienced at least one EE mutation (mean $\Delta w=0.81$) than for adaptive walks that had experienced no EE mutations (mean $\Delta w=0.75$), a difference that is highly significant (Fig. 1g, *P* = 8 × 10^−151^, two-sided Mann–Whitney *U* = 7,390,352, *n* = 6778). More generally, adaptive walks that experienced more EE mutations also experienced a significantly higher increase in mean fitness (Spearman’s *r* = 0.27, *P* = 1.2 × 10^−161^, two-sided, *n* = 10^4^). I also observed that 83.6% of adaptive walks terminated before ten steps had been reached, even though only 66.1% of adaptive walks reached the maximal fitness after ten steps. This means that many adaptive walks become trapped at local fitness peaks. EE mutations may help prevent a population from becoming trapped, because adaptive walks with more EE mutations last longer (Spearman’s *r* = 0.28, *P* = 1.1 × 10^−179^, two-sided, *n* = 10^4^). Indeed, 19.8% (1282/6485) of adaptive walks with at least one EE mutation do not terminate early, whereas the same holds for only about half as many adaptive walks with no EE mutation (10.2%, 359 of 3515), a difference that is highly significant (*P* = 10^−34^, Chi-square = 151.0, 1 df). Analogous simulations for smaller population sizes and without the assumption of weak mutation also show that EE mutations are associated with significant fitness increases (Supplementary Note 1).

### EE mutations are more frequent but have smaller fitness effects in an RNA adaptive landscape

**Fig. 2:**
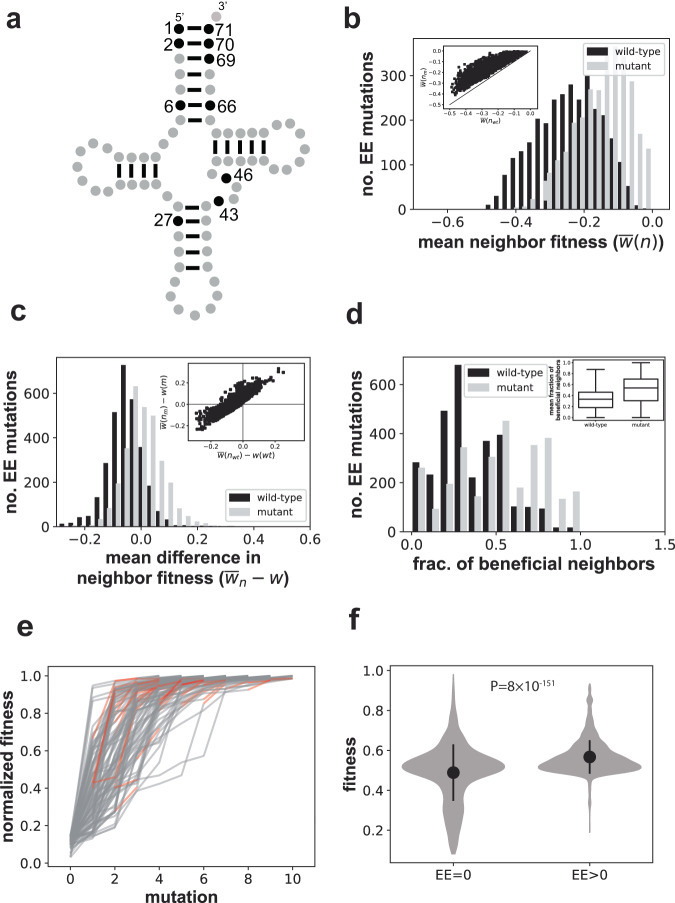
Beneficial EE mutations in an RNA adaptive landscape. **a** Schematic of tRNA secondary structure. Paired bases in stems (helices) are indicated by short straight lines. Black circles indicate those ten positions at which the nucleotide sequence was varied to map the adaptive landscape^26^. The vertical stem on top is the acceptor stem, which contains most of the variable sites. **b** Distribution of the mean fitness of all neighbors of the wild-type ($\bar{w}\left({n}_{{wt}}\right),$ black), as well as of all neighbors of the mutant ($\bar{w}\left({n}_{m}\right),$ gray), for all pairs of neighbors and their beneficial EE mutants in the yeast tRNA fitness landscape^26^. Inset: scatterplot of $\bar{w}\left({n}_{{wt}}\right)$ and $\bar{w}\left({n}_{m}\right)$; diagonal line: $\bar{w}\left({n}_{{wt}}\right)=\bar{w}\left({n}_{m}\right)$. **c** Distribution of the mean fitness of all neighbors of the wild-type adjusted by the fitness of the wild-type ($\bar{w}\left({n}_{{wt}}\right)-w({wt}),$ black), as well as of all neighbors of the mutant adjusted by the fitness of the mutant ($\bar{w}\left({n}_{m}\right)-w(m),$ gray), for all pairs of neighbors and their beneficial EE mutants in the tRNA landscape. Inset: Scatterplot of the same quantities. **d** Distribution of the fraction of beneficial neighbors (neighbors with greater fitness) of the wild-type (black) as well as of the corresponding EE mutant, for all pairs of wild-type sequences and their beneficial EE mutants. Inset: box plot of this fraction (*n* = 2983 wild-type/mutant pairs; box height: interquartile range (IQR), horizontal bar: median; whisker length: 1.5 × IQR). **e** Fitness evolution (vertical axis) during 100 stochastic adaptive walks (horizontal axis: mutational steps) on the RNA landscape. Red edges correspond to EE mutations that occur during an adaptive walk**. f** The fitness difference attained after step three of 10^4^ adaptive walks between walkers that experienced no (EE = 0) and at least one (EE > 0) EE mutations is highly significant (*P* = 2.2 × 10^−39^, two-sided Mann–Whitney *U* = 10,237,878, *n* = 4109). Circles and vertical bars show means and one standard deviation. Fitness values for (**e**, **f**) are normalized to the interval (0,1). Source data are provided as a Source Data file.

I next studied the fitness landscape of an arginine-CCU transfer RNA (tRNA) from the yeast *Saccharomyces cerevisiae* (Fig. 2a). Previous work measured the fitness of 4176 tRNA variants that differ at one or more of 10 nucleotide positions, with two or three variable nucleotides per position that occur naturally in seven yeast species^26^. The authors expressed a library of these variants from a centromeric yeast plasmid in a *S. cerevisiae* strain from which the native (single-copy) gene *HSX1*, which encodes the tRNA, had been deleted. To quantify fitness, the authors deep-sequenced the library before and after selection in an environment where the tRNA is essential for growth (Supplementary Methods)^26^. This procedure allowed them to estimate the fitness of individual variants by estimating their pre-and post-selection sequencing read counts. Data are reported as logarithmically transformed ratios of post-selection read counts of the focal variant and the reference tRNA, that is, the tRNA encoded by the *S. cerevisiae* genome. On this logarithmic measurement scale, *w(g) > 0 (* < *0)* means that genotype *g* grows faster (more slowly) than the *S. cerevisiae* genotype. Fitness values for the 4176 tRNA variants range between −0.86 and 0.09. Although this range differs from that of the protein landscape, I note that the measurement scale (a logarithmically transformed read count ratio) is comparable between the two landscapes (Supplementary Methods)^27^. Except where otherwise mentioned, I use the originally reported fitness scale.

In the RNA landscape, 5.7% of mutations (2983 of 52672) are beneficial and EE-enhancing. For these beneficial EE mutations, Fig. 2b shows histograms of the mean fitness of the neighbors of the wild-type (black) as well as of the mutant (gray). As for proteins, the neighbors of EE mutants tend to have higher fitness than those of the wild-type. Indeed, for all beneficial EE mutations, $\bar{w}\left({n}_{m}\right)=$ −0.15 compared to $\bar{w}\left({n}_{{wt}}\right)=$ −0.25, a difference that is highly significant (*P* = 3.5 × 10^−297^, two-sided Mann–Whitney *U* = 1,998,287, *n* = 2983). Figure 2c shows the histograms of the mean fitness of the wild-type’s neighbors adjusted for the fitness of the wild-type ($\bar{w}\left({n}_{{wt}}\right)-w\left({wt}\right)$), as well as the analogous quantity for the mutants ($\bar{w}\left({n}_{m}\right)-w\left(m\right)$). The right shift of the mean fitness distribution persists with this adjustment. More specifically, averaged over all beneficial EE mutations $\bar{w}\left({n}_{{wt}}\right)-w\left({wt}\right)$ = −0.05 and $\bar{w}\left({n}_{m}\right)-w\left(m\right)$ = +0.005, a difference that is again highly significant (*P* = 5.3 × 10^−241^, two-sided Mann–Whitney *U* = 224,935, *n* = 2983). The positive (albeit small value) of $\bar{w}\left({n}_{m}\right)-w\left(m\right)$ shows that at least some neighbors of mutants are typically beneficial. Indeed there are 968 (32.4%) of beneficial EE mutations whose neighbors are on average beneficial ($\bar{w}\left({n}_{m}\right)-w\left(m\right)$ > 0), whereas the same mutations in the wild-type are on average deleterious ($\bar{w}\left({n}_{{wt}}\right)-w\left({wt}\right)$ < 0, inset of Fig. 2c, upper left quadrant). Figure 2d shows the distribution of the fraction of beneficial neighbors both for the wild-type and each corresponding EE mutant. This distribution is again shifted towards a higher fraction of beneficial mutation in the neighborhood of EE mutations. Overall, 34.3% of wild-type neighbors are on average beneficial, whereas 50.9% of EE mutant neighbors are (Fig. 2d, inset), an increase of 48% (*P* = 1.9 × 10^−145^, two-sided Mann–Whitney *U* = 2,741,913, *n* = 2983). In addition, 76.4% of beneficial EE mutants (2279) have more beneficial neighbors than their wild-type ancestors. In sum, like in the protein landscape, the neighbors of beneficial EE mutations are also more likely to be beneficial. Deleterious EE mutations (7.0% of all mutations) share their main properties with beneficial EE mutations (Supplementary Fig. 2).

As in the protein landscape, beneficial EE mutations are more rare in low-fitness regions of the RNA landscape, but wild-types of intermediate fitness are most likely to give rise to EE-enhancing mutations (Supplementary Fig. 3c). Another noteworthy difference to the protein landscapes is that beneficial EE mutations themselves tend to cause a significantly lower fitness increase (mean $\Delta w$=0.04) than beneficial non-EE mutations (mean $\Delta w$=0.11; *P* < 10^−297^, two-sided Mann–Whitney *U* = 14,619,198, *n*_*1*_ = 2983, *n*_*2*_ = 23,336).

When examining the mutations that enhance evolvability to the greatest extent, I found that they all occur in the tRNA acceptor stem (Fig. 2a). They fall into few categories and tend to stabilize the tRNA secondary structure, for example by creating a G–C base from a mismatched base pair or from a G–U base pair (Supplementary Table 2). Thus, in a genetic background that confers greater RNA structural stability, subsequent mutations may be less deleterious and more likely to be beneficial.

### Adaptive walks with EE mutations also lead to higher fitness gains in the RNA landscape

As in the protein landscape, I first studied 10^4^ stochastic adaptive walks that started out from randomly chosen genotypes within the bottom 5% of the fitness distribution, but with the effective population size of yeast (*N* = 7.8 × 10^6^)^53^. Figure 2e shows the evolutionary trajectories of a sample of 100 of these walks, with EE mutational steps shown in red. Most adaptive walks rapidly reach a fitness plateau, but the landscape appears less navigable than the protein landscape. It takes six instead of just four steps to reach a median fitness that exceeds 90% of the maximally possible fitness, and even after ten steps, the median fitness reached by 10^4^ adaptive walks is $\bar{w}=0.92$, 8% below the maximum. Figure 2e suggests that only a small fraction of adaptive walks overcomes a suboptimal fitness plateau. Indeed, among 10^4^ adaptive walks, none reach the maximal fitness within ten steps, and only 6% reached a fitness within 5% of the maximum. Relatedly, many more adaptive walks (9576 of 10,000) terminate before 10 steps than in the protein landscape (8359 of 10,000).

As in the protein landscape, I study the role of EE mutations after the third step of the adaptive walks. After this step, the difference between the maximum and the minimum fitness among all walks still covers 56% of the total fitness range. Adaptive walks that experience at least one EE mutation had reached a fitness of $\bar{w}=0.51$ after the third step, 7.0% higher than adaptive walks that experience no EE mutations ($\bar{w}=0.48$), a highly significant difference (Fig. 2f; *P* = 2.2 × 10^−39^, two-sided Mann–Whitney *U* = 10,237,878, *n* = 4109). More generally, adaptive walks that experience more EE mutations also experience a significantly higher increase in mean fitness (Spearman’s *r* = 0.15, *P* = 8.7 × 10^−52^, two-sided, *n* = 10^4^). Finally, adaptive walks with more EE mutations are significantly longer (Spearman’s *r* = 0.67, *P* < 10^−297^, two-sided, *n* = 10^4^). Relatedly, adaptive walks with EE mutations are 140 times less likely to become trapped at a local peak than adaptive walks with no EE mutations (5.7% [440/7751] vs. 0.04% [1/2249] of walks with and without EE mutations do not terminate prematurely; *P* = 4.4 × 10^−30^, Chi-Square = 129.8, 1 df). Adaptive walks at smaller population sizes and without the weak-mutation assumption also achieve higher fitness when EE mutations are present (Supplementary Note 1).

### Biological landscapes contain more beneficial EE mutations than expected by chance

The mere existence of EE mutations in biological fitness landscapes is biologically significant if they help populations evolve higher fitness during adaptive evolution, as they do in the landscapes I study. This biological significance is the main focus of my work, but it is also sensible to ask about the statistical significance of EE mutations, i.e., would such mutations be expected to occur “by chance alone” in a suitably defined null model of a “random” fitness landscape? I asked this question for each of the two empirical landscapes by randomly permuting the landscape’s fitness values, determining the incidence of beneficial EE mutations, and repeating this procedure 100 times. In the randomized protein landscape, only 0.0013 ± 8.7 × 10^−6^ (s. dev.)% of mutations (2.3 ± 1.5 mutations) on average are beneficial, a much smaller proportion than the 0.39% of beneficial EE mutations (681 mutations) in the actual protein landscape. Thus, the protein landscape has over 300 times more beneficial EE mutations than expected from the randomized landscape. Also, not a single one of the 100 randomized landscapes has as many beneficial EE mutations as the protein landscape. Thus, the number of EE mutations in the protein landscape is greater than expected by chance alone at a statistical significance of *P* < 0.01. In the randomized RNA landscape, 1.8 ± 0.08 (s. dev.)% of mutations (971 ± 40 mutations) are beneficial and EE, whereas in the RNA landscape, 5.7% of mutations (2983 mutations) are beneficial and EE. In other words, the RNA landscape contains 3.1 times more beneficial EE mutations than expected by chance. This difference is also statistically significant at *P* < 0.01. In sum, both landscapes contain significantly more beneficial EE mutations than expected by chance, and dramatically more so for the protein landscape.

## Discussion

Multiple properties of biological systems have been linked to evolvability^1,4,40,54^. Some of them, such as the shortness of the amino acid motifs recognized by regulatory protein kinases, or the structure of the genetic code^1,40,54,55^, are global organizational properties of life that do not evolve on short evolutionary time scales. This makes them poorly suited to study the evolution of evolvability.

Other, more local properties are better suited, because they can evolve rapidly. Among them is a genotype’s potential to experience mutations that are adaptive (or at least only weakly deleterious). Single EE mutations can affect this potential. They belong in a broader class of mutations that modify the distribution of fitness effects (DFEs) of other mutations. Because knowledge about the DFE is necessary to understand the evolutionary dynamics of populations, techniques to quantify it from empirical data have been developed^50^. DFE modifiers have also been studied theoretically, albeit in other contexts^56,57^. For example, theory predicts that DFE modifiers that render mutations more deleterious may help drive viral populations to extinction^56^.

EE mutations are DFE modifiers that shift the DFE towards greater fitness. In both the protein and the RNA landscape, beneficial (as well as deleterious) EE mutations exist and comprise a small minority of mutations. They increase the mean fitness of other mutations, reduce the deleterious effects of deleterious mutations, and increase the fraction of mutations that are beneficial. In other words, they do not just make maladaptive mutations less maladaptive, but also increase the incidence of adaptive mutations. Some single EE mutations can shift the DFE dramatically toward more beneficial mutations. The occurrence of EE mutations during adaptive evolution can increase the fitness of an evolving population significantly.

A beneficial EE mutation that renders another beneficial mutation even more beneficial provides an example of a positively epistatic (nonadditive) interaction, where the combined effect of two mutations is stronger than that of both mutations individually (Supplementary Methods). Positive epistasis is the exception^58,59^, and negative epistasis is the rule in adaptive landscapes^29,33,38,39,60–62^, which may help explain why EE mutations are rare. Consistent with this observation, most fitness landscapes show a form of negative epistasis known as diminishing returns epistasis^11,31,63–65^, in which beneficial mutations become less beneficial as an evolving population becomes increasingly well-adapted to its environment.

I note that multiple alternative definitions of an EE mutation are conceivable. First, a mutation could be considered EE if it merely helps reduce the deleterious effects of other mutations, even if it does not itself increase the incidence of beneficial mutations. Mutations that render proteins more stable have this effect, and their suppression of deleterious mutation can help enzymes evolve new catalytic activities^21^. Second, one might require that an EE mutation must increase the proportion of subsequent mutations that are beneficial. Many but not all of the EE mutations I identify have this property. Third, one could exclude all neighbors of a wild-type/mutant pair with very low fitness from further analysis, because they may play little role in adaptive evolution. Fourth, one could require that an EE mutation increases the fitness of two, three, and higher mutant neighbors (and not just those in the immediate neighborhood of the mutant). Finally, one could also average neighbor fitness in different ways, or use different measurement scales for fitness. Which definition turns out to be most useful may well depend on the question asked and on the landscape studied.

I focused here on EE mutations that are themselves beneficial, because under the high population sizes that are typically of microbes, deleterious mutations are very unlikely to go to fixation. For example, at *E. coli’s* population size of *N* = 1.8 × 10^8^, a deleterious mutation whose fitness is lower than that of the wild-type by a mere s = 10^−7^ has a negligible probability of *P* = 4.6 × 10^−23^ to go to fixation^66^. However, exceptions may exist. For example, in a long-term *E. coli* evolution experiment, initially deleterious mutations increased a population’s potential to evolve the ability to extract energy from a new carbon source, which allowed these mutations to persist in the population^10^. It is thus important that the main properties of deleterious EE mutations are similar to those of beneficial EE mutations (Supplementary Figs. 1 and 2).

Both the protein and RNA landscapes I study harbor EE mutations, but they differ in many other respects. First, beneficial EE mutations are many times less abundant in the protein landscape (0.39 vs. 5.7%). Second, EE mutations in the protein landscape increase the fitness of other mutations more strongly ($\left(\bar{w}\left({n}_{m}\right)-\bar{w}\left({n}_{{wt}}\right)\right)-\Delta w\,\approx \,0.21$) than in the RNA landscape ($\left(\bar{w}\left({n}_{m}\right)-\bar{w}\left({n}_{{wt}}\right)\right)-\Delta w\,\approx \,0.055$).Third, in the protein landscape the fitness benefit of beneficial EE mutations is similar to that of non-EE mutations. In contrast, in the RNA landscape, the fitness benefit of EE mutations is smaller than that of non-EE mutations. This property may help explain why EE mutations that occur during adaptive walks lead to a smaller fitness increase in the RNA landscape than in protein landscapes. Which, if any, of these differences may be universal differences of protein and RNA landscapes is an exciting question for future work. Answering it will become possible as more such landscapes are being characterized.

The evolutionary dynamics on landscapes with EE mutations may be complex. For example, on landscapes like that of the tRNA (Fig. 2a) the first mutation(s) to be fixed will probably not be EE-enhancing if adaptive evolution starts out in a low-fitness region (Supplementary Fig. 3). The reason is that EE mutations are rare in such regions. Once such an EE mutation becomes fixed, it may increase or decrease the likelihood that further mutations are EE. Furthermore, interactions between mutations may also affect the adaptive benefit of EE mutations. For example, consider a large population with a high mutation rate, in which multiple beneficial clones that compete with each other for fixation occur at the same time. Such clones will be subject to clonal interference^67–71^, which may prevent EE mutations (with a small fitness benefit) from going to fixation and favor non-EE mutations (with a larger fitness benefit). As experimental data on more landscapes become available, the evolutionary dynamics of EE mutations may also become a rewarding study subject.

One limitation of my analysis is that it considers only the small number of variable sites used in mapping the protein and RNA landscape. This is an unavoidable limitation, because it would be impossible to construct a combinatorially complete adaptive landscape for even a short RNA molecule like a tRNA (≈70 nucleotides), an entire protein, or an entire genome. To mitigate this limitation experimentally, one could (i) engineer specific pairs of wild-type molecules (protein or RNA) and their putative EE mutants based on landscape analysis, (ii) subject them to random mutagenesis over the entire length of the molecule, and (iii) measure the (mean) fitness of the randomized variants. If a mutation is truly EE, one would expect that this mean fitness is higher in the EE mutant background. A second limitation is that my quantitative observations may not be directly comparable between landscapes, because of differences in how fitness is reported^26,27^. Such a comparison is not central for my purpose, but where it is, different landscapes may have to be mapped with the same experimental protocols. Third, I only studied the consequences of EE mutations on adaptive evolution in the short term. In the long term, they may have additional consequences. For example, they may increase the mutational load of a population.

In sum, my analysis shows that EE mutations exist in both protein and RNA landscapes, and that they can help increase fitness during adaptive evolution. Do such EE mutations exist in all proteins or RNA molecules? Do they differ in their abundance among different kinds of such molecules? Do they create evolutionary contingencies, where populations that encounter such mutations take different evolutionary paths than those that do not. Do such mutations enhance evolvability across different environments? Can they help make qualitatively new phenotypes accessible to Darwinian evolution? As more and larger landscapes are being characterized, these and other questions will open many avenues for research on the evolution of evolvability.

## Methods

For both the protein and RNA landscape, I first identified all pairs of sequences that are (one-mutant) neighbors of each other, i.e., they differ only in a single residue, and arbitrarily designated one as the “wild-type” (*wt*) and the other as the mutant (*m*). I then classified the mutant as beneficial if $\varDelta w=w\left(m\right)-w\left({wt}\right)\, > \,0,$ and as deleterious if $\varDelta w\, < \,0.$ I do not explicitly study neutral EE mutations here, because such mutations cannot be reliably identified for large populations with today’s technology to measure fitness, and the number of 1-mutant neighbor pairs where both fitness measurements happen to be exactly equal is so small as to be negligible (38 pairs or 0.02% of all pairs for the protein dataset and 34 or 0.06% for the RNA dataset). I then determined all ${k}_{{wt}}$ one-mutant neighbors of the ${wt}$ sequence, excluding those sequences that differ from the ${wt}$ at the same residue at which $m$ differs from it. For these neighbors, I computed the mean fitness $\bar{w}\left({n}_{{wt}}\right)$ and its standard deviation $\sigma \left({n}_{{wt}}\right)$. I repeated this calculation for the ${k}_{m}$ neighbors of the mutant $m$ to obtain their mean fitness $\bar{w}\left({n}_{m}\right)$ and its standard deviation $\sigma \left({n}_{m}\right)$. I note that ${k}_{{wt}}\, \ne \, {k}_{m}$ may hold, because for a small fraction of genotypes, fitness data is not available. The maximally possible number of neighbors of any genotype in the RNA landscape equals *k* = 6 × 1 + 4 × 2 = 14.

With this information in hand, I then tested the null hypothesis that the mutation ${wt}\to m$ is *not* evolvability-enhancing. To this end, I used a two-sided one-sample *t* test of the null hypothesis that $\bar{w}\left({n}_{m}\right)-\bar{w}\left({n}_{{wt}}\right)\le \varDelta w$, which is equivalent to asking whether $\bar{w}\left({n}_{m}\right)-w\left(m\right)\le \bar{w}\left({n}_{{wt}}\right)-w\left({wt}\right)$. If the null hypothesis is rejected, i.e., if the difference $\bar{w}\left({n}_{m}\right)-\bar{w}\left({n}_{{wt}}\right)$ is significantly greater than the difference $\varDelta w$ in fitness between the wild-type and the mutant$,$ the mutation is evolvability-enhancing, according to inequality (2). To compute the standard deviation of $\bar{w}\left({n}_{m}\right)-\bar{w}\left({n}_{{wt}}\right)$, which is necessary for the *t* test, I took advantage of the fact that the standard deviation of the difference of two independent random variates *x* and *y* (with individual standard deviations ${\sigma }_{x}$ and ${\sigma }_{x}$) is given by ${\sigma }_{y-x}=\sqrt{{\sigma }_{x}^{2}+{\sigma }_{y}^{2}}$. Thus, the standard deviation of $\bar{w}\left({n}_{m}\right)-\bar{w}\left({n}_{{wt}}\right)$, computes as ${\sigma }^{2}(\varDelta \bar{w}(n))=\sqrt{{\sigma }^{2}\left({n}_{{wt}}\right)+{\sigma }^{2}\left({n}_{m}\right)}$. I used ${{{\min }}}({k}_{{wt},}{k}_{m})$, i.e., the smaller of the two numbers of neighbors of the wild-type and mutant genotype$,$ as the degrees of freedoms for this *t* test. This procedure renders the *t* test conservative. I performed such *t* tests for all beneficial mutations in both data tests, and used a Benjamini–Hochberg correction^72^ with a false discovery rate (FDR) of 0.01 to correct for multiple testing. Supplementary Methods describe an analogous procedure to identify deleterious EE mutations.

The published protein landscape data contains only information on the fitness of each amino acid sequence variant of the antitoxin, but not on the DNA genotype encoding this variant. Due to the structure of the genetic code^73^, not all 20 amino acids can be reached from one another through single-point mutations. In my analysis of this landscape, I admitted only those genotypes as 1-mutant neighbors that differ in a single amino acid, and where the two differing amino acids (i.e., amino acid ${a}_{1}$ in genotype 1 and ${a}_{2}$ in genotype 2) are accessible from each other through a single-point mutation. This means that there must exist at least one codon ${c}_{1}$ for ${a}_{1}$, and at least one codon ${c}_{2}$ for ${a}_{2}$, such that codons ${c}_{1}$ and ${c}_{2}$ differ by only a single nucleotide. The Supplementary Methods provide further information on fitness measurements.

To model adaptive evolution on the two landscapes, I focus on a weak-mutation scenario^18,51,52^, which is appropriate, because I study the evolution of small parts of single genes in which mutations are much rarer than in whole genomes. The scenario requires that the population mutation rate, i.e., the product of (effective) population size *N* and per-nucleotide mutation rate μ is small, i.e., *Nμ* < 1. For example, in *E. coli*, with a per-nucleotide mutation rate of *μ* = 2 × 10^−10^ and an effective population size of *N* = 1.8 × 10^8^, the per-generation population mutation rate in the three amino acid (nine nucleotide) region of the gene I study is given by 9*Nμ* = 0.32, and only a fraction of these mutations would be non-synonymous, creating a new amino acid sequence^53^. Likewise, in the yeast *S. cerevisiae*, μ = 2.6 × 10^−10^, and *N* = 7.8 × 10^6^, such that 10Nμ = 0.02 for the ten-nucleotide RNA landscape I study^53^). In the weak-mutation scenario, a population is monomorphic most of the time until a mutation occurs that goes to fixation. Most mutations do so rapidly relative to the waiting time for the next mutation that goes to fixation, and after such a fixation event the population again remains monomorphic until this next mutation occurs, and so on. In other words, adaptive evolution can be modeled as if it was an adaptive walk of a single genotype changing through point mutations.

To model this adaptive walk, I took advantage of Kimura’s formula for the probability $${p}_{{fix}}$$ that a mutation goes to fixation in a haploid population under the influence of selection and drift, $${p}_{{fix}}=(1-{e}^{-2s})$$\$$(1-{e}^{-2{Ns}})$$ (Equation 3.11 of ref. ^66^). Here, *N* is the effective population size and *s* denotes the selection coefficient of the mutant, i.e., its difference in fitness from the pre-mutated (wild-type) state. If s > 0 (s < 0) the mutation is beneficial (deleterious). At the large population sizes of *E. coli* and yeast, deleterious mutations are very unlikely to go to fixation, and the same holds for neutral mutations $$({s=0,p}_{{fix}}=1/N)$$, such that beneficial mutations dominate the evolutionary dynamics.

To simulate adaptive evolution on each of the two adaptive landscapes, I first constructed a graph whose nodes are the genotypes of the landscape. Two nodes are connected by a directed edge if they are one-mutant neighbors. For a given population size *N*, I then computed for each edge the fixation probability ${p}_{{fix}}$ of the corresponding mutational step, where I calculated the selection coefficient *s* of the respective mutation as the difference in fitness between the genotype after and before mutation. I note that in this graph, for each “forward” edge connecting two neighboring genotypes ${g}_{i}$ and ${g}_{j}$ (and its ${p}_{{fix}}$) there is a corresponding “backward” edge connecting ${g}_{j}$ and ${g}_{i}$, together with an associated probability ${p}_{{fix}}$.

Starting from any one genotype *g* on an adaptive landscape, I simulated each step of an adaptive walk as follows. I divided (“normalized”) the fixation probability ${p}_{{fix}}$ of each neighbor of *g* by the sum of the fixation probabilities of all neighbors. Subsequently, I chose one of these neighbors at random, such that each neighbor had an equal likelihood to be chosen, and generated a (pseudo)random number on the interval (0,1). If this random number was smaller than the (normalized) fixation probability of the mutational step leading to this neighbor, the corresponding mutation goes to fixation, i.e., the adaptive walk progresses by one step towards this neighbor. If not, I repeated the procedure, choosing another neighbor, and another random number, and so on, until I had found a successful fixation event.

The above normalization of fixation probabilities is necessary to render the adaptive walks computationally feasible. The reason is that the fixation probabilities of all neighbors of a genotype *g* can be very small if the fitness of these neighbors is very similar to that of *g*. In this case, waiting times for a successful fixation event become excessive. The normalization helps avoid this problem while preserving the important relative magnitude of the fixation probabilities. I note that as a result of this normalization, information on the time scale of adaptive evolution is lost.

With this procedure, I performed 10^4^ adaptive walks, each with a maximum of 10 mutational steps. The initial genotype for each adaptive walk I chose at random (with uniform distribution) from the genotypes within the lowest 5% (the 5th percentile) of the fitness distribution on the landscape. Whenever such an adaptive walk reached a genotype *g* for which all neighbors had a smaller fitness than *g* and a fixation probability of ${p}_{{fix}}$ = 0, I considered a local adaptive peak in the landscape to have been reached. In this case, I terminated the adaptive walk and recorded the number of steps from the starting genotype to the local peak.

To explore the consequences of relaxing the assumption of weak mutation, I also simulated adaptive evolution under a simple model of clonal interference, a phenomenon that occurs at high population mutation rates and that leads to the co-occurrence of multiple beneficial mutations that compete with each other for fixation^67–71^. In this model, only the fittest mutation goes to fixation. I modeled this scenario through a “greedy” adaptive walk, in which the most beneficial neighbor of the current genotype always goes to fixation. For the display of adaptive walks, I normalized fitness values of both landscapes to the interval (0,1) to facilitate visual comparison between RNA and protein landscapes (Figs. 1f, g and 2e, f). To test for differences in the location of statistical distributions, I used two-sided Mann–Whitney *U* tests implemented in scipy (version 1.9.1) of Python (version 3.8.5).

### Reporting summary

Further information on research design is available in the Nature Portfolio Reporting Summary linked to this article.

### Supplementary information

Supplementary Information

Reporting Summary

### Source data

Source Data

## Data Availability

All landscape data analyzed here has been obtained from previous publications. Specifically, RNA landscape data are publicly available as Supplementary Table S1 from the Supplementary Information section of ref. ^26^. Protein data is taken from ref. ^27^ and publicly available through accession number GSE153897 from the NCBI Gene Expression Omnibus database. The protein structure shown in Fig. 1b is based on data in protein database (pdb) file 5CEG (10.2210/pdb5CEG/pdb) Source data are provided with this paper.

## Code availability

Scripts to analyze the landscape data are publicly available at https://github.com/andreas-wagner-uzh/EE_mutations.
