# Supplementary material for: Evolvability-enhancing mutations in the fitness landscapes of an RNA and a protein
Source: Nat Commun. 2023 Jun 19;14:3624. doi: 10.1038/s41467-023-39321-8 (PMC10279741; doi:10.1038/s41467-023-39321-8)
Supplement: Supplementary file 1 — Supplementary Information [file 41467_2023_39321_MOESM1_ESM.pdf]

Supplementary Online Material to

Evolvability-enhancing mutations in the fitness landscapes of an RNA and a protein

Andreas Wagner

Supplementary Methods

Defining deleterious EE mutations. In general, it can be useful to define and study deleterious evolvability-enhancing (EE) mutations, because (weakly) deleterious mutations can occasionally spread through a population via genetic drift and can thus affect evolvability [1-3]. However, I note that at the large population sizes I study here, even weakly deleterious mutations are unlikely to go to fixation. For example, at the population size of *E.coli* (N=1.8×10^8), the fixation probability of a mutation that reduces fitness by as little as s=10^{-7} equals p=4.6×10^{-23}. Such mutations thus play little role in the evolutionary dynamics I study here.

Applying the definition of a beneficial EE mutation (inequality 2 of the main text) to a deleterious EE mutation ($\Delta w = w(m) - w(wt) < 0$) has an undesirable consequence that is best illustrated with an example. Consider a mutation m that (i) reduces fitness by some amount, e.g., Δw=s<0, and that (ii) causes all other point mutations in its background to reduce their fitness by only half that amount on average, compared to the wild-type background, i.e., $\bar{w}(n_m) - \bar{w}(n_{wt}) = \frac{s}{2} < 0$. According to requirement (2), mutation m would enhance evolvability. But in the background of m, point mutations would still have lower fitness than in the wild-type, on average. Thus, arguably, the mutation has not increased the likelihood that subsequent mutations produce variation that is adaptive.

To avoid this problem, I apply a stronger criterion to define evolvability-enhancement for deleterious mutations: I require that even in the background of the deleterious mutation m, point mutations have a higher fitness on average than in the wild-type background, i.e.,

$$\bar{w}(n_m) - \bar{w}(n_{wt}) > 0, \qquad \text{(S1)}$$

a condition that is identical to that for neutral mutations.

Together, inequalities 1, 2, and S1 motivate the following definition. A mutation is evolvability-enhancing if

$$\bar{w}(n_m) - \bar{w}(n_{wt}) > \max(0, \Delta w) \qquad \text{(S2)}$$

This condition is equivalent to requiring that m shows *on average* positive epistasis with the mutations leading to its neighbors (see *EE mutations and positive epistasis* below).

To identify deleterious EE mutations requires testing the null hypothesis that a specific deleterious mutation $wt \rightarrow m$ ($\Delta w < 0$) is *not* evolvability-enhancing, To this end I used a procedure exactly analogous to that described in Methods to identify beneficial EE mutations in both the protein and the RNA landscapes. Specifically, I used a two-sided one-sample t-test of the null hypothesis that $\bar{w}(n_m) - \bar{w}(n_{wt}) \leq 0$, which is equivalent to asking whether $\bar{w}(n_m) \leq \bar{w}(n_{wt})$. If the null-hypothesis is rejected, the mutation is evolvability-enhancing by the criterion of inequality (S1). I calculated the variance of $\bar{w}(n_m) - \bar{w}(n_{wt})$ and the degrees of freedom of the t-test analogously to that for beneficial mutations described in Methods. I applied the test to all deleterious mutations. I corrected for multiple testing with the Benjamini-Hochberg method at a false discovery rate (FDR) of 0.01[4].

EE mutations and positive epistasis. Consider a wild-type genotype *ab*, two single mutants *Ab* and aB, as well as the double mutant AB. Then epistatic interactions between these mutations can be described through the expression

$$\varepsilon = w(ab) + w(AB) - w(Ab) - w(aB),$$

i.e., the mutations $a \rightarrow A$ and $b \rightarrow B$ interact epistatically if $\varepsilon \neq 0$. More specifically, epistasis is positive (negative) if $\varepsilon > 0$ ($\varepsilon < 0$) [5-8]. I will now translate this widely used notation into the language of this paper, and show that the criteria for an EE mutation from inequalities (2) and (S1) are formally equivalent to those for positive epistasis when applied to single mutations in the neighborhood of a wild-type *wt* and a mutant *m*.

Consider a wild-type genotype *wt* (corresponding to genotype *ab* above), and an EE-enhancing single mutation *m* (corresponding to genotype *Ab*). Consider now a single neighbor $n_{wt,i}$ (different from *m*) of the wild-type (corresponding to genotype *aB*), as well as the corresponding neighbor of the mutant $n_{m,i}$ (corresponding to genotype *AB*). Then the above expression becomes

$$\varepsilon = w(wt) + w(n_{m,i}) - w(m) - w(n_{wt,i}) = w(n_{m,i}) - w(n_{wt,i}) - \Delta w$$

Case 1. The mutation m is beneficial or neutral *(*$\Delta w = w(m) - w(wt) \geq 0$*)*: In this case, if the mutation *m* fulfills the criterion of inequality (2) for an EE mutation, when applied only to this neighbor $n_{wt,i}$ and $n_{m,i}$, i.e., $w(n_{m,i}) - w(n_{wt,i}) > \Delta w$, it follows that $\varepsilon > 0$, i.e., epistasis is positive.

Case 2. The mutation m is *deleterious (*$\Delta w < 0$*)*: In this case, if the mutation *m* fulfills the criterion of inequality (S1) for an EE mutation, when applied only to this neighbor $n_{wt,i}$ and $n_{m,i}$, i.e., $w(n_{m,i}) - w(n_{wt,i}) > 0$. It follows again that $\varepsilon > 0$, i.e., epistasis is positive.

In either case, if $\varepsilon > 0$ for all mutations $n_{wt,i}$ and $n_{m,i}$ in the neighborhood of the wild-type and the mutant, then a more stringent condition than that of inequalities (2) and (S1) holds, i.e., the mutation *m* increases the relative fitness of *all* neighbors. Because inequalities (2) and (3) require only that $\varepsilon > 0$ when averaged over all neighbors, these conditions amount to requiring positive epistatic interactions on average.

Fitness data for the protein landscape. The reference antitoxin protein ParD3 from *Mesorhizobium opportunistum* binds to its cognate toxin protein ParE3[9,10]. In the absence of such binding, for example through a loss-of-function mutation in ParD3, ParE3 inhibits cell growth [11]. Toxin-antitoxin pairs like these are useful tools to study how the specificity of protein-protein interactions can affect fitness. Three amino acids D61, K64 and E80 at the interface between the toxin and the antitoxin are crucial to determine the specificity of the interaction of the ParD3 antitoxin with its cognate toxin ParE3, because specific mutations at all three positions eliminate this specificity and lead to low fitness [9]. In a previous study 20^3=8000 plasmid-encoded antitoxin variants were expressed in a population of *E. coli,* together with a plasmid that expressed the

cognate toxin ParE3 from an inducible promoter. The authors subjected the library to Illumina deep-sequencing before and after 10 hours of growth under toxin expression. As a measure of the fitness of each variant relative to all other variants, the authors used the logarithmically transformed ratio of the number of reads of each variant after and before selection, normalized by the optical density of the *E.coli* population, and relative to the logarithmically transformed ratio of all other sequence reads before and after selection [9,10]. Raw fitness scores were then normalized such that (i) the *M. opportunistum* (DKE) antitoxin sequence had a fitness of one, and (ii) the median fitness of variants with a stop-codon had a fitness of zero. After this normalization, fitness values in the library ranged from -0.21 to 1.02, i.e., some library members have lower fitness than nonsense variants or higher fitness than the *M. opportunistum* genotype.

Fitness data for the RNA landscape. A previous study that mapped the fitness landscape of arginine-CCU transfer RNA (tRNA) from the yeast *Saccharomyces cerevisiae* relied on naturally occurring variants of this gene that occur in six yeast species closely related to *S. cerevisiae*[12]. These variants occur at ten positions of the tRNA, and comprise two different nucleotides at each of six of the ten positions, as well as three different nucleotides at the remaining four positions. The authors created a combinatorially complete library of all 5,184 (=$2^6 \times 3^4$) combinations of these variants, and expressed this library from a centromeric yeast plasmid in a *S. cerevisiae* strain from which the native (single-copy) gene *HSX1*, which encodes the tRNA, had been deleted. They quantified the fitness of yeast cells expressing each library variant in an environment where the tRNA is essential for growth (synthetic complete [SC] medium lacking histidine, 37ºC, 1M NaCl). More specifically, they subjected the library of tRNA variants to Illumina HiSeq 125bp paired-end sequencing both before and after 6.5 generations of growth in the selective environment. For each library variant, the authors report pre-and post-selection sequencing read counts that are normalized by the total number of reads and the optical density of the populations in which the library was expressed. From these values they estimated the fitness of a genotype relative to the *Saccharomyces cerevisiae* reference genotype as a logarithmically (base e) –transformed ratio of post-selection read counts of the focal variant and the *S. cerevisiae* genotype. In this ratio, read-counts are additionally weighted by the inverse of their measurement variance, which is determined from the number of reads in two pre-selection replicate libraries, and three post-selection replicates derived from each pre-selection replicate. This procedure yielded fitness data on 4,176 tRNA variants (Table S1 of ref. 12), which range between -0.86 and 0.09.

Supplementary Note 1: Adaptive walks at varying population sizes, as well as 'greedy' adaptive walks, also lead to higher fitness if EE mutations occur in them.

Protein Landscape. The adaptive walks in the main text are based on an assumption of large population size. At smaller population sizes drift is stronger, selection is weaker, and EE enhancing mutations may thus have little impact on the evolutionary dynamics. However, adaptive walks at two much smaller population sizes (N=10^5 and N=10^2) show that this is not the case. Specifically, at N=10^5 and N=10^2, evolving populations that experience at least one EE mutation acquire 7.8 percent and 5.9 percent higher fitness, differences that are statistically significant (N=10^5: P=2.5×10^{-147}, two-sided Mann-Whitney U=7520270, n=6718; N=10^2: P=1.5×10^{-126}, two-sided Mann-Whitney U= 7929673, n=6613). Second, adaptive walks with more EE mutations also tend to reach higher fitness at the smaller population sizes (Spearman's r=0.26 and r=0.24 for N=10^5 and N=10^2, respectively, P<2.1×10^{-135}, two-sided). Third, adaptive walks with more EE mutations are less likely to become trapped at a fitness peak for N=10^5 (Spearman's r=0.28, P=5.1×10^{-180}, two-sided). Relatedly, a significantly smaller proportion of adaptive walks with at least one EE mutation does not terminate early (9.7 percent vs. 20.2 percent; P=5.3×10^{-41}, Chi-square=179.8, 1 df). This no longer holds for N=10^2, where none of the 10^4 adaptive walk terminates prematurely. The reason is that drift in these small population is sufficiently strong that escape from fitness peaks is always possible.

In a next analysis, I explored the consequences of relaxing the assumption of weak mutation. If this assumption is violated, multiple beneficial mutations can occur in the same population and lead to clonal interference, where these beneficial mutations compete with each other for fixation. In this case, the mutation with highest fitness is most likely to go to fixation [18-22]. I modeled this scenario through a 'greedy' adaptive walk, in which the most beneficial mutation inevitably goes to fixation at each step. Here again, adaptive walks with at least one EE mutation reach significantly higher fitness (P=6.4×10^{-27}, two-sided Mann-Whitney U=10878766, n_1=4612, n_2=5388), although the difference (2.9 percent) is smaller than in the absence of clonal interference. The association between the number of EE mutations and this increase in fitness is still highly significant but weak (Spearman's r=0.07, P<8.8×10^{-14}, two-sided). Greedy adaptive walks are more likely to be terminating early. For example, every single one among 10,000 greedy adaptive walks had terminated by step 7, whereas only 3,008 of 10,000 stochastic adaptive walks (N=10^8) had done so at that time point, and 1641 stochastic adaptive walks had not yet terminated by step 10. Longer greedy adaptive walks tend to harbor significantly more EE mutations (Spearman's r=0.69, P<10^{-297}, two-sided).

RNA Landscape. At two much smaller population sizes (N=10^4 and N=10^2), evolving populations that experience at least one EE mutation acquire 7.8 percent and 3.4 percent higher fitness in the tRNA landscape, differences that are statistically significant (N=10^4: P=7.5×10^{-58}, two-sided Mann-Whitney U= 9362829, n=3652; N=10^2: P=9.6×10^{-16}, two-sided Mann-Whitney U= 10148766, n=3418). In addition, adaptive walks with more EE mutations also tend to reach higher fitness at the smaller population sizes (Spearman's r=0.21 and r=0.12 for N=10^4 and N=10^2, respectively; P<2×10^{-34}, two-sided). Adaptive walks with more EE mutations are also less likely to become trapped prematurely at a fitness peak for N=10^4 (Spearman's r=0.23,

$P<1.1\times10^{-119}$, two-sided). Relatedly, a significantly smaller proportion of adaptive walks with at least one EE mutation do not terminate early (32 percent vs. 86.1 percent; $P<10^{-297}$, Chi-square=1532.9, 1 df). This no longer holds for $N=10^2$, where none of the 10^4 adaptive walk terminates prematurely.

I also explored the consequences of clonal interference through greedy adaptive walks. Greedy adaptive walks in which at least one EE mutation occurred reached 7.5 percent higher fitness than such walks in which no EE mutation occurred, a difference that is highly significant ($P=3.9\times10^{-36}$, two-sided Mann-Whitney U=10402617, n_1=4215, n_2=5785). The association between the number of EE mutations and this increase in fitness is still highly significant but weak (Spearman's r=0.14, $P=3.3\times10^{-43}$, two-sided). Greedy adaptive walks with more EE mutations also tend to terminate significantly later (Spearman's r=0.61, $P<10^{-297}$, two-sided).

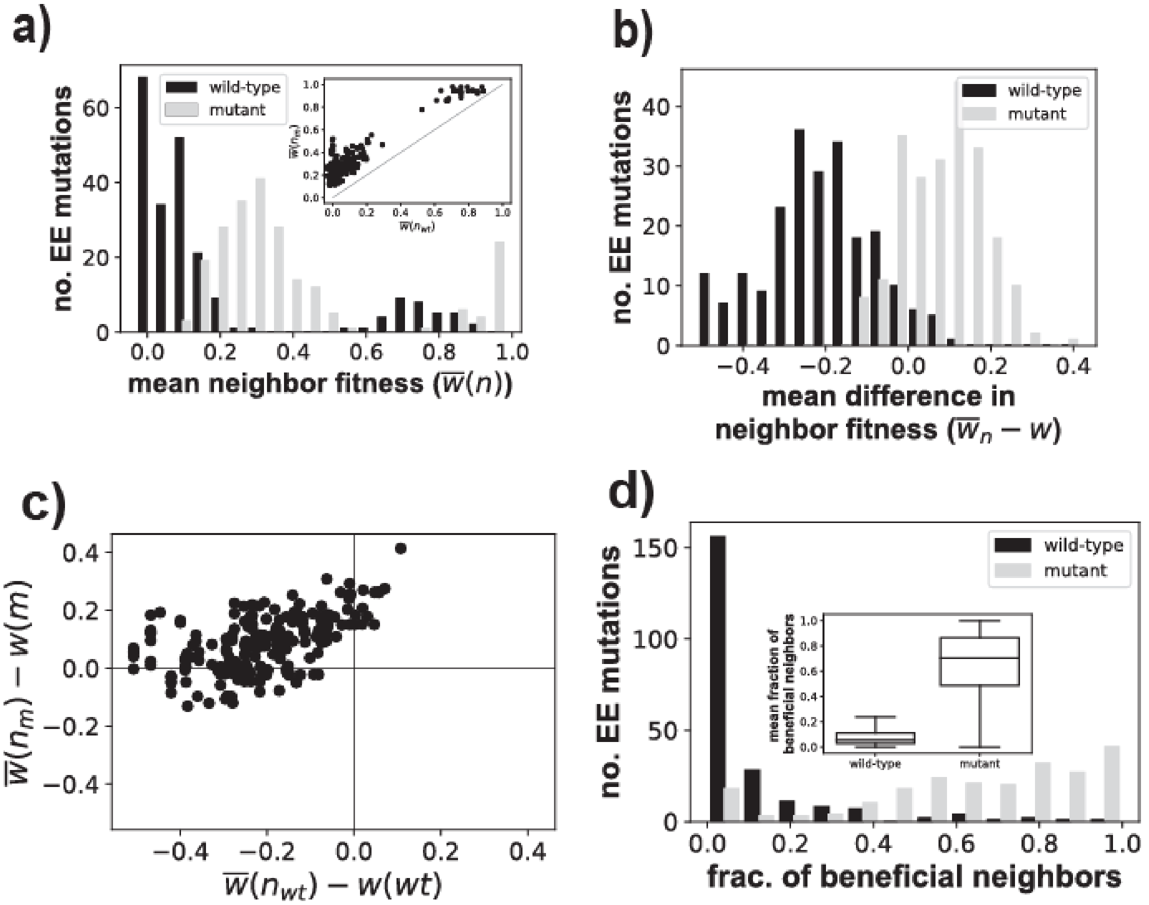

Figure S1: Deleterious EE mutations in the protein adaptive landscape. **a)** Only 0.13 percent (221) of all mutations in the protein landscape are deleterious and evolvability-enhancing. Distribution of the mean fitness of all neighbors of the wild-type ($\overline{w}(n_{wt})$, black), as well as of all neighbors of the mutant ($\overline{w}(n_m)$, gray), for all pairs of neighbors and their deleterious EE mutants in the ParD3 antitoxin fitness landscape of ref. 9. Similarly to beneficial EE mutations (Figure 1), the grey distribution is shifted to the right, i.e., the mean fitness of the neighbors of the mutant is substantially higher than that of the neighbors of the wild-type. When averaged over all deleterious EE mutations, the mean fitness of the neighbors of the mutant $\overline{w}(n_m) = 0.39$, which is more than twice as high as the mean fitness of the wild-type $\overline{w}(n_{wt}) = 0.17$, a difference that is highly significant ($P=1.8\times10^{-37}$, two-sided Mann-Whitney U=7243, n=221). Inset: scatterplot of $\overline{w}(n_{wt})$ and $\overline{w}(n_m)$; diagonal line: $\overline{w}(n_{wt}) = \overline{w}(n_m)$. **b)** Distribution of the mean fitness of all neighbors of the wild-type adjusted by the fitness of the wild-type ($\overline{w}(n_{wt}) - w(wt)$, black), as well as of all neighbors of the mutant adjusted by the fitness of the mutant

($\overline{w}(n_m) - w(m)$, gray), for all pairs of neighbors and their beneficial EE mutants in the protein landscape. Averaged over all deleterious EE mutations $\overline{w}(n_{wt}) - w(wt)$=-0.21 and $\overline{w}(n_m) - w(m)$=0.008, a difference that is again highly significant (P=5.7×10^{-65}, two-sided Mann-Whitney U=1565, n=221). The positive sign of $\overline{w}(n_m) - w(m)$ shows that deleterious EE mutations do not just reduce the deleterious effects of deleterious mutations, but can also increase a genotype's potential to bring forth beneficial mutations. **c)** Scatterplot of $\overline{w}(n_{wt}) - w(wt)$ and $\overline{w}(n_m) - w(m)$. There are 155 EE mutations (upper left quadrant, 70.1 percent of all deleterious mutations) whose neighbors are on average beneficial ($\overline{w}(n_m) - w(m)$>0), whereas the same mutations in the wild-type are on average deleterious ($\overline{w}(n_{wt}) - w(wt)$<0). **d)** Distribution of the fraction of beneficial neighbors (neighbors with greater fitness) of the wild-type (black), as well as of the corresponding mutant, for all pairs of wild-type sequences and their beneficial EE mutants. Inset: box plot of this fraction (n=221 wild-type/mutant pairs; box height: interquartile range (IQR), horizontal bar: median; whisker length: 1.5×IQR). Overall, 10.5 percent of wild-type neighbors are on average beneficial, whereas 64.6 percent of EE mutant neighbors are (inset), an increase that is statistically highly significant (P=1.1×10^{-52}, two-sided Mann-Whitney U=2940, n=221).

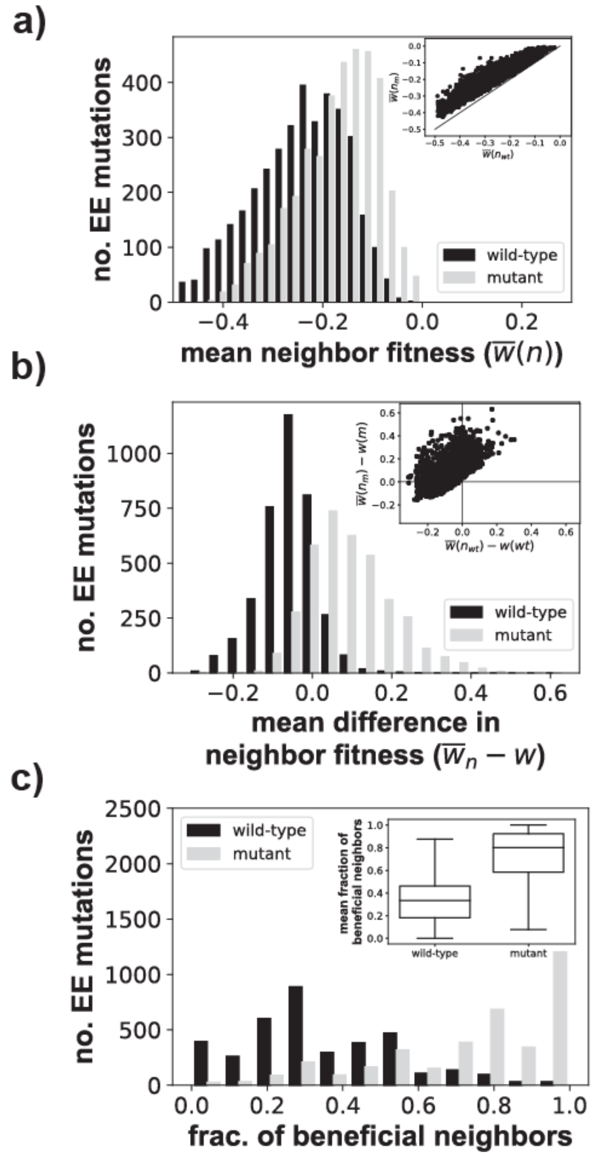

Figure S2: Deleterious EE mutations in the RNA adaptive landscape. 7.0 percent (3702/52672) of mutations in the RNA landscape are deleterious EE mutations. **a)** Distribution of the mean fitness of all neighbors of the wild-type ($\overline{w}(n_{wt})$, black), as well as of all neighbors of the mutant ($\overline{w}(n_m)$, gray), for all pairs of neighbors and their deleterious EE mutants. Inset: scatterplot of $\overline{w}(n_{wt})$ and $\overline{w}(n_m)$; diagonal: $\overline{w}(n_{wt}) = \overline{w}(n_m)$. For all deleterious EE mutations, $\overline{w}(n_m) =$ -0.17 compared to $\overline{w}(n_{wt}) =$ -0.24, a difference that is highly significant ($P=5.1\times10^{-232}$, two-sided Mann-Whitney U= 3861538, n =3702). **b)** Distribution of the mean fitness of all neighbors of the wild-type adjusted by the fitness of the wild-type ($\overline{w}(n_{wt}) - w(wt)$, black), as well as of all neighbors of the mutant adjusted by the fitness of the mutant ($\overline{w}(n_m) - w(m)$,

gray), for all pairs of neighbors and their deleterious EE mutants in the RNA landscape. Averaged over all deleterious EE mutations $\bar{w}(n_{wt}) - w(wt)$=-0.06 and $\bar{w}(n_m) - w(m)$=+0.09, a difference that is highly significant ($P<10^{-297}$, two-sided Mann-Whitney U= 13568389, n =3702). Inset: scatterplot of $\bar{w}(n_{wt}) - w(wt)$ against ($\bar{w}(n_m) - w(m)$. There are 2360 (63.7 percent) of deleterious EE mutations whose neighbors are on average beneficial ($\bar{w}(n_m) - w(m)$>0), whereas the same mutations in the wild-type are on average deleterious ($\bar{w}(n_{wt}) - w(wt)$<0 (upper left quadrant of inset).**c)** Distribution of the fraction of beneficial neighbors (neighbors with greater fitness) of the wild-types (black) as well as of all corresponding deleterious EE mutants (grey). Inset: box plot of this fraction (n=3702 wild-type/mutant pairs; box height: interquartile range (IQR), horizontal bar: median; whisker length: 1.5×IQR). 33.7 percent of wild-type neighbors are on average beneficial, whereas 74.1 percent of EE mutant neighbors are (inset), an increase of 119 percent that is statistically highly significant ($P<10^{-297}$, two-sided Mann-Whitney U= 1581304, n=3702). In addition, 95.9 percent of EE mutants (3550) have a greater number of beneficial neighbors than their wild-type ancestors. As in the protein landscape, the neighbors of deleterious EE mutations are thus also more likely to be beneficial.

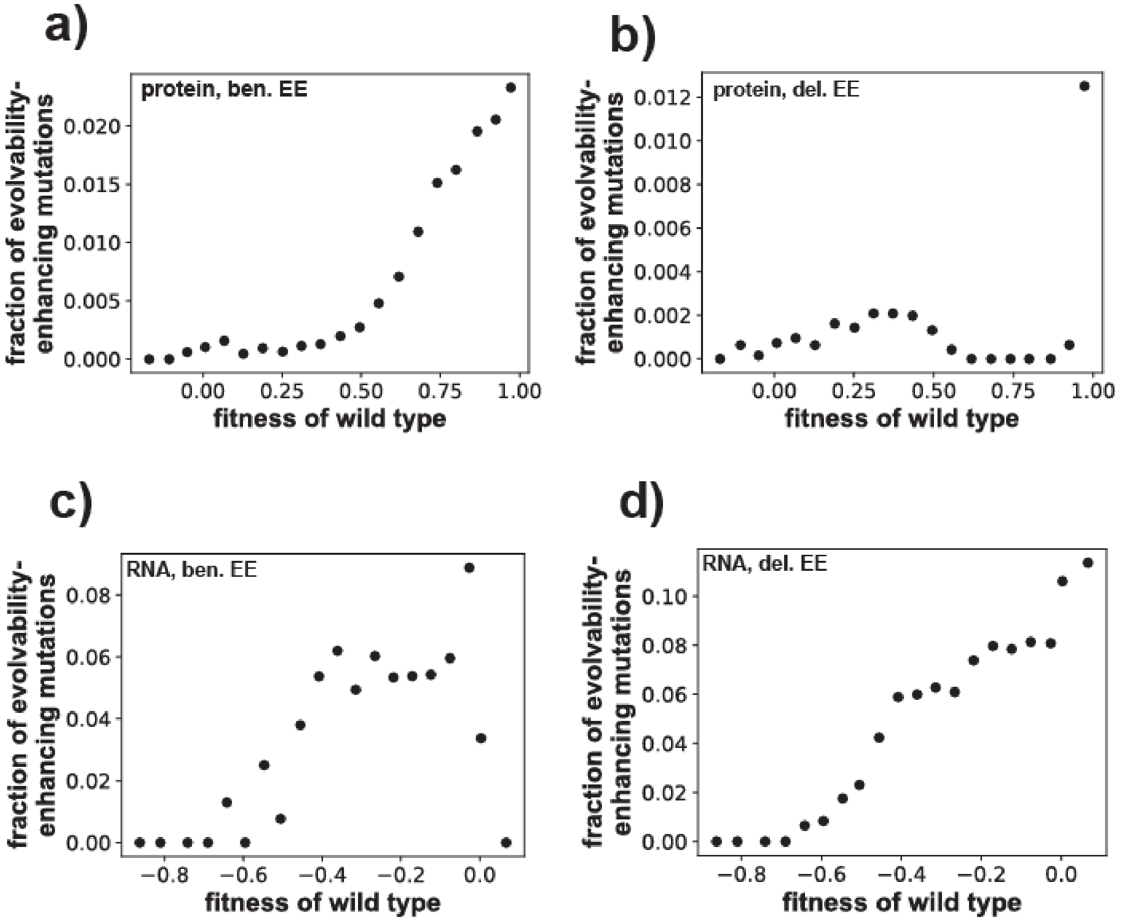

Figure S3: **The incidence of EE mutations depends on the fitness of the wild-type genotype.** To generate the data in this figure, I grouped wild-type genotypes according to their fitness into 20 equally wide bins that cover the entire range of fitness values. The horizontal axis shows the mean fitness of wild-type genotypes in each bin. The vertical axis shows the fraction of beneficial EE mutations (panel a and c) and of deleterious EE mutations (panel b and d) among all mutations in each bin, for both the protein landscape (panel **a** and **b**) and the RNA landscape (panel **c** and **d**). Statistical associations between wild-type fitness and the fraction of EE mutation are a) Spearman's rank correlation r=0.96, $P=3.7\times10^{-11}$ (two-sided); b) r=-0.1, P=0.69 (two-sided); c) r=0.62, P=0.034 (two-sided); d) r=0.99, $P=1.7\times10^{-17}$ (two-sided), sample size n=20 for all four associations. The statistical outlier in the highest fitness bin of panel b is based on few (29) deleterious EE mutations of 2319 wild-type genotypes in the bin. The fitness scales differ between the protein and RNA data, because the original data were reported on different scales[9,12]. I note that the fraction of EE mutations in each bin of each landscape is generally small. The occurrence of a small proportion of EE mutations even in high fitness regions of a landscape is consistent with a prediction from simple theoretical models that positive (synergistic) epistasis, which favors evolvability [8], can exist even close to a fitness peak [23]. In

both the protein and RNA landscape, deleterious EE mutations cause a significantly smaller fitness loss on average than deleterious non-EE mutations (protein: Δw=-0.08 for deleterious EE mutations and Δw=-0.17 for deleterious non-EE mutations on average; P=6.5×10^{-24}; two-sided Mann-Whitney U=13465685, n_1=221, n_2=87536; RNA: Δw=-0.08 for deleterious EE mutations and Δw=-0.11 for deleterious non-EE mutations; P=8.4×10^{-85}, two-sided Mann-Whitney U= 50226506, n_1=3702, n_2=22617).

wt	m	aa(wt)	aa(m)	w(wt)	w(m)	w(m)-w(wt)	mean w(n_{wt})	mean w(n_m)	mean w(n_m)-mean w(n_{wt})	f_{ben}(n_{wt})	f_{ben}(n_m)
DKP	DKQ	P	Q	0.8	0.9878	0.1874	0.1664	0.9489	0.7825	0	0.0526
DKP	DKL	P	L	0.8	0.9623	0.1619	0.1664	0.9403	0.7739	0	0.1053
MNY	MKY	N	K	0.408	0.8996	0.4921	0.1282	0.8938	0.7656	0.0263	0.6579
DKP	DKH	P	H	0.8	0.9799	0.1795	0.1664	0.9311	0.7647	0	0.0263
DKP	DKT	P	T	0.8	0.977	0.1766	0.1664	0.9305	0.7641	0	0
DKP	DKA	P	A	0.8	0.9674	0.167	0.1664	0.9266	0.7602	0	0.0263
GFG	DFG	G	D	0.094	0.5449	0.4505	0.086	0.8403	0.7543	0.3684	0.8649
KNK	KKK	N	K	0.35	0.818	0.468	0.1156	0.8668	0.7512	0.0263	0.7895
DKP	DKS	P	S	0.8	0.9901	0.1897	0.1664	0.9111	0.7447	0	0
NNC	NKC	N	K	0.272	0.9188	0.6466	0.161	0.905	0.744	0.2105	0.5
YFG	DFG	Y	D	0.055	0.5449	0.4899	0.1017	0.8403	0.7386	0.75	0.8649
DKP	DKR	P	R	0.8	0.9801	0.1797	0.1664	0.8874	0.7211	0	0
GYE	DYE	G	D	0.448	1.0002	0.5523	0.2261	0.9471	0.7211	0.2105	0.1053
INF	IKF	N	K	0.398	0.9049	0.5071	0.1826	0.8998	0.7172	0.0526	0.6316
VFG	DFG	V	D	0.069	0.5449	0.4757	0.1254	0.8403	0.7149	0.8158	0.8649
MNE	MKE	N	K	0.386	0.9618	0.5759	0.2234	0.9294	0.706	0.1842	0.3684
CNC	CKC	N	K	0.354	0.7602	0.406	0.0896	0.7929	0.7033	0.0263	0.6842
MNM	MKM	N	K	0.369	0.8775	0.5085	0.1958	0.8917	0.6959	0.1053	0.7895
QNI	QKI	N	K	0.575	0.9073	0.3319	0.2041	0.898	0.6939	0.0556	0.5405
LNI	LKI	N	K	0.325	0.9518	0.6266	0.2328	0.9255	0.6927	0.2105	0.1351
**Table S1**											

Table S1: Top 20 EE mutations in the ParD3 antitoxin fitness landscape, sorted by $\bar{w}(n_{wt}) - \bar{w}(n_m)$. The first and second column from the left indicate the wild-type and the mutant genotype, respectively, at positions 61, 64, and 80 of the ParD3 antitoxin, written as a three character string in the standard single letter amino acid alphabet. The *M. opportunistum* reference genotype is 'DKE', i.e., D61, K64, and E80. Columns 3 and 4 show the amino acids that distinguish the wild-type and the mutant. Columns 5-7 show the fitness $w(wt)$ of the wild-type, the fitness $w(m)$ of the mutant, and the difference in their fitness $\Delta w = w(m) - w(wt)$, as discussed in the main text. Columns 8-10 show the mean fitness $\bar{w}(n_{wt})$ of the neighbors of the wild-type, the mean fitness $\bar{w}(n_m)$ of the neighbors of the mutant, and their difference $\bar{w}(n_m) - \bar{w}(n_{wt})$. Columns 11 shows the fraction $f_{ben}(n_{wt})$ of neighbors of the wild-type that are beneficial, i.e., they have a higher fitness than the wild-type. Column 12 shows the fraction $f_{ben}(n_{mt})$ of neighbors of the mutant that are beneficial, i.e., they have a higher fitness than the mutant. Colors group mutations into the following categories. Green: In 7 of the top 20 mutations, the wild-type genotype is DKP, and the mutation replaced the proline at the third position of the DKP motif with a different amino acid (A, H, L, Q, R, S, or T). The wild-type genotype differs from the *M. opportunistum* genotype (DKE) only in this proline, which often affects protein phenotypes strongly, because it disrupts α-helices. Indeed, proline reduces fitness to a greater extent than all other amino acid changes at the third position of the *M. opportunistum* reference (see Figure 2E of ref. 9), even though this fitness reduction is modest (Table S1). Changing the proline to one of the other seven amino acids, however, increases the fitness of the resulting genotypes to a value w>0.96, much closer to the *M. opportunistum* DKE. Thus, the EE mutations are beneficial because proline reduces fitness more strongly than other amino acid

changes. Note that for these seven mutations, the neighbors of the DKP wild-type have much lower fitness ($\overline{w}(n_{wt}) = 0.17$) than the DKP mutant itself ($w(DKP) = 0.8$, Table S1). In contrast, the neighbors of the mutants have much higher mean fitness ($0.89 \leq \overline{w}(n_m) \leq 0.95$), which is not much below the fitness of the mutants (w>0.96). Thus, strong synergistic epistasis exists between the proline at the third position and the mutations at the other two positions. Yellow: In these 9 mutations the amino acid asparagine (N) at the second position mutates to the *M. opportunistum* lysine (K), thus decreasing the distance to the *M. opportunistum* DKE genotype by one step. Here the wild-type has much lower fitness than in the first class of mutations ($0.27 \leq w(wt) \leq 0.58$), and the mutations cause a much greater increase in fitness ($0.33 \leq \Delta w \leq 0.65$). The broad range of this increase comes from the diversity of the nine wild-type genotypes, which share little except the N at position two. The neighbors of the wild-type have much lower fitness ($0.09 \leq \overline{w}(n_{wt}) \leq 0.23$), than the wild-type itself, whereas the neighbors of the mutant have substantially elevated fitness ($0.8 \leq \overline{w}(n_m) \leq 0.93$), which lies within five percent of the mutant. This indicates again strong synergistic epistasis between the amino acid N defining the wild-type and the other amino acids in the genotype. The fraction of beneficial neighbors of the mutant (0.14-0.79) is much greater than the fraction of beneficial neighbors of the wild-type (0.03-0.21) than for the first (green) mutation category. Blue: In 4 of the mutations amino acids G, V, or Y in the first position mutate into D. Thus, as in the second (yellow) category, all mutations create a genotype that is one step closer to the *M. opportunistum* genotype DKE. The fitness increase caused by the mutations is substantial ($0.45 \leq \Delta w \leq 0.55$), and while the fitness of the wild-type neighbors is very low ($0.09 \leq \overline{w}(n_{wt}) \leq 0.23$), the fitness of the neighbors of the mutants is much higher ($0.84 \leq \overline{w}(n_m) \leq 0.95$). For three of the four neighbors, it even lies above that of the mutant ($\overline{w}(n_m) = 0.84$ vs. $\overline{w}(m) = 0.55$). The fraction of beneficial neighbors of the mutant (0.11-0.86) is greater than the fraction of beneficial neighbors of the wild-type (0.21-0.82). In sum, the beneficial mutations that are most strongly EE enhancing fall into s few categories characterized by changes either from or to specific amino acids, and strong epistasis. Unfortunately, the biophysical causes of this epistasis are currently not understood.

wt	m	pos	nt(wt)	nt(m)	w(wt)	w(m)	w(m)-w(wt)	mean w(n_{wt})	mean w(n_m)	mean w(n_m)-mean w(n_{wt})	$f_{ben}(n_{wt})$	$f_{ben}(n_m)$
GUUUCCAGAU	GUUUCCAGAC	71	U	C	-0.3334	-0.2637	0.0697	-0.4432	-0.151	0.2922	0	0.6667
GUUCACAGUU	GUUCACAGUC	71	U	C	-0.4079	-0.2908	0.1171	-0.4076	-0.1248	0.2828	0.5	0.9167
GGUUCUAGUU	GGUUCUAGUC	71	U	C	-0.3855	-0.3571	0.0284	-0.4341	-0.1839	0.2502	0.4615	0.8182
GUUUCCCGGC	GUGUCCCGGC	6	U	G	-0.4009	-0.2354	0.1655	-0.3893	-0.1449	0.2444	0.6364	0.75
GGGCACUGUU	GGGCACUGUC	71	U	C	-0.3811	-0.2855	0.0956	-0.384	-0.1416	0.2425	0.4615	0.8462
AGUCAUUAGU	ACUCAUUAGU	2	G	C	-0.2163	-0.0224	0.1939	-0.3374	-0.0962	0.2412	0.1667	0.3333
GUACACUGUU	GUACACUGUC	71	U	C	-0.4473	-0.335	0.1123	-0.3708	-0.13	0.2408	0.6667	1
AGACCUUGGU	ACACCUUGGU	2	G	C	-0.2057	-0.0138	0.192	-0.365	-0.1244	0.2406	0	0
AGUCCCUGGU	ACUCCCUGGU	2	G	C	-0.3681	-0.3445	0.0236	-0.4457	-0.2069	0.2388	0	0.8889
GGACACAGUU	GGACACAAUU	69	G	A	-0.31	-0.1229	0.1871	-0.3782	-0.1405	0.2377	0.4444	0.5385
AGAUCCUAGC	ACAUCCUAGC	2	G	C	-0.3372	-0.2997	0.0375	-0.3394	-0.1022	0.2371	0.4	1
AUUUCCAAGC	ACUUCCAAGC	2	U	C	-0.3297	-0.2988	0.0309	-0.319	-0.0846	0.2344	0.6667	1
GGGCACUGUU	GGGCACUGGU	70	U	G	-0.3811	-0.2045	0.1766	-0.4051	-0.172	0.2331	0.4167	0.75
AUUCCUAGGC	ACUCCUAGGC	2	U	C	-0.2585	-0.2498	0.0087	-0.2902	-0.0586	0.2316	0.4444	1
AUGCAUUGUU	AUGCAUUGUC	71	U	C	-0.3532	-0.162	0.1911	-0.3849	-0.1541	0.2308	0.4167	0.5385
AGGCAUCAGU	ACGCAUCAGU	2	G	C	-0.1656	0.0009	0.1665	-0.2508	-0.0206	0.2302	0.25	0.0833
AUACAUUGAU	AUACAUUAAU	69	G	A	-0.0213	0.0009	0.0222	-0.3115	-0.0824	0.2291	0	0
GUACAUUGUU	GUACAUUGUC	71	U	C	-0.3502	-0.2024	0.1478	-0.3492	-0.1216	0.2276	0.4167	0.8462
GCUCAUAGUU	GCUCAUAGUC	71	U	C	-0.1852	-0.1429	0.0423	-0.3224	-0.0952	0.2272	0.2222	0.7
AUGCAUUGAC	AUGCAUUAAC	69	G	A	-0.2018	-0.118	0.0839	-0.3039	-0.0772	0.2268	0.5	0.7692
Table S2												

Table S2: Top 20 EE mutations in the tRNA fitness landscape, sorted by $\bar{w}(n_{wt}) - \bar{w}(n_m)$. The first and second column from the left indicate the wild-type and the mutant genotype, respectively, at all ten positions of the tRNA sequence that are variable in the studied landscape, i.e., positions 1, 2, 6, 27, 43, 46, 66, 69, 70, 71 (Figure 1A) [12]. The genotypes at these positions are shown as concatenated strings of length ten. Column 3 indicates at which of the ten positions the wild-type and mutant genotype differ. Columns 4 and 5 show the nucleotides that distinguish the wild-type and the mutant. Columns 6-8 show the fitness $w(wt)$ of the wild-type, the fitness $w(m)$ of the mutant, and the difference in their fitness $\Delta w = w(m) - w(wt)$. Columns 9-11 show the mean fitness $\bar{w}(n_{wt})$ of the neighbors of the wild-type, the mean fitness $\bar{w}(n_m)$ of the neighbors of the mutant, and their difference $\bar{w}(n_m) - \bar{w}(n_{wt})$. The evolvability benefit of the top 20 beneficial EE mutations is more modest and covers a narrower range of values ($0.23 \leq \bar{w}(n_m) - \bar{w}(n_{wt}) \leq 0.29$) than their counterparts in the protein landscape (Table S1). Column 12 shows the fraction $f_{ben}(n_{wt})$ of neighbors of the wild-type that are beneficial, i.e., they have a higher fitness than the wild-type. Column 13 shows the fraction $f_{ben}(n_m)$ of neighbors of the mutant that are beneficial. Most mutations in the table are likely to stabilize tRNA secondary structure, which is a prerequisite for tRNA function. In general, RNA secondary structure is stabilized by contributions that include those of Watson-Crick base pairs (G-C or A-U), slightly less favorable wobble (G-U) base pairs, and the stacking of adjacent base pairs in an RNA stem (helix)[24-28]. The table's mutations can be grouped into the following color-coded categories, whose members create specific kinds of base pairs. Green: These comprise eight U71C mutations, each of which transforms a wobble G-U base pair between the tRNA acceptor stem into a G-C base pair. Possibly because of the increased stability conveyed by the G-C base pair, each mutation increases fitness ($0.03 \leq \Delta w \leq 0.19$). The fitness of the neighbors of the wild-type (U71) is much lower ($-0.44 \leq \bar{w}(n_{wt}) \leq -0.32$) than those of the mutant (G71, $-0.18 \leq \bar{w}(n_{mt}) \leq -0.1$), i.e., in the mutant background, mutations have on average greater fitness ($0.23 \leq \bar{w}(n_m) - \bar{w}(n_{wt}) \leq 0.29$). In addition, for each of the eight mutations, the proportion

of beneficial neighbors of the mutant is greater than for the wild-type. Blue: This category comprises 7 mutations at position 2 of the acceptor stem, i.e., five G2C mutations and two U2C mutations (blue in Table S2). The base at position 2 is paired with that at position 70, which has genotype G70 in all 7 genotypes. Thus, all of the mutations create a C-G Watson-Crick base pair, five of them from a G-G mismatch, and the remaining two from a U-G wobble base pair. The fitness increase for all seven mutations ($0.01 \leq \Delta w \leq 0.19$) is very similar to that of the first class of mutations. The seven mutations cause an increase in the fitness of their neighbors that varies little, and is similar to that for the first class of mutations ($0.23 \leq \bar{w}(n_m) - \bar{w}(n_{wt}) \leq 0.24$). For five of the seven mutations, the fraction of beneficial neighbors is greater in the mutant background than in the wild-type background. Grey: This category comprises three G69A changes. The base at position 69 pairs with that at position 3 of the acceptor loop, which is conserved (U69) in the tRNA sequences considered [12]. Thus, each of the mutations creates a U-A Watson-Crick base pair from a U-G wobble base pair. Yellow and purple: These comprise two unique mutations, a U6G mutations that creates a G-C base pair from a U-C mismatch in the acceptor stem, and a U70G mutation that creates a G-G mismatch from a G-U wobble base pair. Despite the destabilizing nature of the last mutation, it causes a fitness increase of $\Delta w = 0.18$. (Beneficial mutations that cause the loss of a Watson-Crick base pair are not unprecedented, see e.g, Figure S6 of ref. 12).With the exception of this last mutation, 19 out of the top 20 EE mutations may help stabilize the tRNA secondary structure, insofar as Watson-Crick base pairs provide greater stability than wobble base pairs or mismatched base pairs[27]. They suggest that in a genetic background that confers greater RNA structural stability, subsequent mutations are less deleterious and more likely to be beneficial.

References

1 Phillips, K. N., Castillo, G., Wünsche, A. & Cooper, T. F. Adaptation of Escherichia coli to glucose promotes evolvability in lactose. *Evolution* **70**, 465-470 (2016).

2 Díaz Arenas, C. & Cooper, T. F. Mechanisms and selection of evolvability: experimental evidence. *FEMS Microbiology Reviews* **37**, 572-582 (2013).

3 Woods, R. J. *et al.* Second-order selection for evolvability in a large Escherichia coli population. *Science* **331**, 1433-1436 (2011).

4 Benjamini, Y. & Hochberg, Y. Controlling the false discovery rate - a practical and powerful approach to multiple testing. *Journal of the Royal Statistical Society Series B - Methodological* **57**, 289-300 (1995).

5 Poelwijk, F. J., Kiviet, D. J., Weinreich, D. M. & Tans, S. J. Empirical fitness landscapes reveal accessible evolutionary paths. *Nature* **445**, 383-386 (2007).

6 Poelwijk, F. J., Tănase-Nicola, S., Kiviet, D. J. & Tans, S. J. Reciprocal sign epistasis is a necessary condition for multi-peaked fitness landscapes. *Journal of Theoretical Biology* **272**, 141-144 (2011).

7 Aguilar-Rodriguez, J., Payne, J. A. & Wagner, A. 1000 empirical adaptive landscapes and their navigability. *Nature Ecology and Evolution* **1**, 0045 (2017).

8 Payne, J. L. & Wagner, A. The causes of evolvability and their evolution. *Nature Reviews Genetics* **20**, 24-38 (2019).

9 Lite, T.-L. V. *et al.* Uncovering the basis of protein-protein interaction specificity with a combinatorially complete library. *Elife* **9** (2020).

10 Aakre, C. D. *et al.* Evolving new protein-protein interaction specificity through promiscuous intermediates. *Cell* **163**, 594-606 (2015).

11 Muthuramalingam, M., White, J. C., Murphy, T., Ames, J. R. & Bourne, C. R. The toxin from a ParDE toxin-antitoxin system found in Pseudomonas aeruginosa offers protection to cells challenged with anti-gyrase antibiotics. *Molecular microbiology* **111**, 441-454 (2019).

12 Domingo, J., Diss, G. & Lehner, B. Pairwise and higher-order genetic interactions during the evolution of a tRNA. *Nature*, 1 (2018).

13 Desai, M. M., Fisher, D. S. & Murray, A. W. The speed of evolution and maintenance of variation in asexual populations. *Current Biology* **17**, 385-394 (2007).

14 van Nimwegen, E., Crutchfield, J. & Huynen, M. Neutral evolution of mutational robustness. *Proceedings of the National Academy of Sciences of the U.S.A.* **96**, 9716-9720 (1999).

15 Gillespie, J. H. Molecular evolution over the mutational landscape. *Evolution*, 1116-1129 (1984).

16 Lynch, M. *et al.* Genetic drift, selection and the evolution of the mutation rate. *Nature Reviews Genetics* **17**, 704-714 (2016).

17 Kimura, M. *The neutral theory of molecular evolution*. (Cambridge University Press, 1983).

18 de Visser, J. A. G. & Rozen, D. E. Limits to adaptation in asexual populations. *Journal of evolutionary biology* **18**, 779-788 (2005).

19 Wilke, C. O. The speed of adaptation in large asexual populations. *Genetics* **167**, 2045-2053 (2004).

20 Lang, G. I. *et al.* Pervasive genetic hitchhiking and clonal interference in forty evolving yeast populations. *Nature* **500**, 571-574 (2013).
21 Gerrish, P. J. & Lenski, R. E. The fate of competing beneficial mutations in an asexual population. *Genetica* **102**, 127-144 (1998).
22 de Visser, A. J. G. M., Zeyl, C. W., Gerrish, P. J., Blanchard, J. L. & Lenski, R. E. Diminishing returns from mutation supply rate in asexual populations. *Science* **283**, 404-406 (1999).
23 Greene, D. & Crona, K. The changing geometry of a fitness landscape along an adaptive walk. *PLoS computational biology* **10**, e1003520 (2014).
24 Schuster, P. Prediction of RNA secondary structures: from theory to models and real molecules. *Reports on Progress in Physics* **69**, 1419 (2006).
25 Hofacker, I. L. *et al.* Fast folding and comparison of RNA secondary structures. *Monatshefte für Chemie* **125**, 167-188 (1994).
26 Zuker, M. & Sankoff, D. RNA secondary structures and their prediction. *Bulletin of Mathematical Biology* **46**, 591-621 (1984).
27 Freier, S. M. *et al.* Improved free-energy parameters for predictions of RNA duplex stability. *Proceedings of the National Academy of Sciences* **83**, 9373-9377 (1986).
28 Varani, G. & McClain, W. H. The G· U wobble base pair. *EMBO reports* **1**, 18-23 (2000).
