# Supplementary material for: Evolvability-enhancing mutations in the fitness landscapes of an RNA and a protein
Source: Nat Commun. 2023 Jun 19;14:3624. doi: 10.1038/s41467-023-39321-8 (PMC10279741; doi:10.1038/s41467-023-39321-8)
Supplement: Supplementary file 2 — Reporting Summary [file 41467_2023_39321_MOESM2_ESM.pdf]

nature portfolio

Corresponding author(s): Andreas Wagner

Last updated by author(s): May 4, 2023

# Reporting Summary

Nature Portfolio wishes to improve the reproducibility of the work that we publish. This form provides structure for consistency and transparency in reporting. For further information on Nature Portfolio policies, see our Editorial Policies and the Editorial Policy Checklist.

## Statistics

For all statistical analyses, confirm that the following items are present in the figure legend, table legend, main text, or Methods section.

| n/a | Confirmed | |
|---|---|---|
| ☐ | ☒ | The exact sample size (*n*) for each experimental group/condition, given as a discrete number and unit of measurement |
| ☐ | ☒ | A statement on whether measurements were taken from distinct samples or whether the same sample was measured repeatedly |
| ☐ | ☒ | The statistical test(s) used AND whether they are one- or two-sided<br>*Only common tests should be described solely by name; describe more complex techniques in the Methods section.* |
| ☒ | ☐ | A description of all covariates tested |
| ☐ | ☒ | A description of any assumptions or corrections, such as tests of normality and adjustment for multiple comparisons |
| ☐ | ☒ | A full description of the statistical parameters including central tendency (e.g. means) or other basic estimates (e.g. regression coefficient) AND variation (e.g. standard deviation) or associated estimates of uncertainty (e.g. confidence intervals) |
| ☐ | ☒ | For null hypothesis testing, the test statistic (e.g. *F*, *t*, *r*) with confidence intervals, effect sizes, degrees of freedom and *P* value noted<br>*Give P values as exact values whenever suitable.* |
| ☒ | ☐ | For Bayesian analysis, information on the choice of priors and Markov chain Monte Carlo settings |
| ☒ | ☐ | For hierarchical and complex designs, identification of the appropriate level for tests and full reporting of outcomes |
| ☒ | ☐ | Estimates of effect sizes (e.g. Cohen's *d*, Pearson's *r*), indicating how they were calculated |

*Our web collection on statistics for biologists contains articles on many of the points above.*

## Software and code

Policy information about availability of computer code

| | |
|---|---|
| Data collection | Data was downloaded from publicly available sources, which required no commercial or custom-made software. |
| Data analysis | Data was analyzed with custom-made python scripts (version 3.8.5), which are publicly available on github (https://github.com/andreas-wagner-uzh/EE_mutations). |

For manuscripts utilizing custom algorithms or software that are central to the research but not yet described in published literature, software must be made available to editors and reviewers. We strongly encourage code deposition in a community repository (e.g. GitHub). See the Nature Portfolio guidelines for submitting code & software for further information.

## Data

Policy information about availability of data

All manuscripts must include a data availability statement. This statement should provide the following information, where applicable:

- Accession codes, unique identifiers, or web links for publicly available datasets
- A description of any restrictions on data availability
- For clinical datasets or third party data, please ensure that the statement adheres to our policy

This paper used only publicly available experimental data and created no experimental data on its own. All its data has been obtained from previous publications and is publicly available through the supplementary information section of refs. 26,27. Source data are provided as a Source Data file. The protein structure shown in Figure 1b is based on data in protein data base file 5CEG (https://doi.org/10.2210/pdb5CEG/pdb)

## Human research participants

Policy information about studies involving human research participants and Sex and Gender in Research.

| | |
|---|---|
| Reporting on sex and gender | not applicable |
| Population characteristics | not applicable |
| Recruitment | not applicable |
| Ethics oversight | not applicable |

Note that full information on the approval of the study protocol must also be provided in the manuscript.

# Field-specific reporting

Please select the one below that is the best fit for your research. If you are not sure, read the appropriate sections before making your selection.

☐ Life sciences ☐ Behavioural & social sciences ☒ Ecological, evolutionary & environmental sciences

For a reference copy of the document with all sections, see nature.com/documents/nr-reporting-summary-flat.pdf

# Ecological, evolutionary & environmental sciences study design

All studies must disclose on these points even when the disclosure is negative.

| | |
|---|---|
| Study description | The study analyzes the occurrence of mutations that increase the average fitness of other mutations in two large, publicly available data sets that measured the fitness of thousands of variants of an E.coli protein and a yeast tRNA. |
| Research sample | No research 'samples' were obtained in this paper, but its data was taken from two publicly available sources. The first is a collection of more than 8000 variant genotypes of the ParD3 protein, whose fitness had been previously measured by other authors, and was obtained from the supplementary material of Lite et al. (Elife 2020). The second is a collection of more than 4000 variant genotypes of a yeast tRNA whose fitness had been measured by other authors, and was obtained from the supplementary material of Domingo et al., (Nature 2020). Complete citations to both sources are provided in the paper. |
| Sampling strategy | This is an analysis of existing, publicly available data data, which was analyzed exhaustively. No sampling was involved, with the following exception. To start 10'000 independent adaptive random walks, genotypes within the bottom 10% of the fitness distribution of all (>4000) genotypes were chosen. Although these random walk starting points were sampled at random (with replacement), the total number of genotypes in the bottom 10 percent is only of the order of 10^2, such that each genotype was chosen multiple times as a starting point. This sampling strategy was dictated by the limited size of the adaptive landscape studied. It was sufficient for the purpose of this study, because it revealed highly significant differences between adaptive walks in the presence and absence of EE mutations. |
| Data collection | Data was collected by the sole author from the supplementary information of two publications cited above and in the paper. |
| Timing and spatial scale | Not applicable. This is not an experimental study but an analysis of existing data. |
| Data exclusions | No data was excluded from the analysis. |
| Reproducibility | Because this study uses experimental data obtained by others, it would be inappropriate to comment on the reproducibility of that data. That being said, the previously published fitness measurements that this paper works with have been made in at least two biological replicates by the authors of the previous work, showed a high correlation between these replicates, and are thus in this sense reproducible. However, to distinguish the minute fitness differences that are visible to selection in an organism like E. coli with large population sizes, one would need more than thousand-fold replication of such measurements, which is cost-prohibitive with today's technology. |
| Randomization | This is not an experimental study but an analysis of existing data obtained by others. Grouping of organisms into treatment and control groups , where randomization would be important, does not take place in this study. Thus, randomization is not applicable to this study. |
| Blinding | This is not an experimental study but an analysis of existing data obtained by others. No research subjects, animals, or plants are subdivided into treatment and control groups, where blinding of the observer and possibly the subjects would be necessary or advisable. Blinding is not applicable to this study. |

Did the study involve field work? ☐ Yes ☒ No

# Reporting for specific materials, systems and methods

We require information from authors about some types of materials, experimental systems and methods used in many studies. Here, indicate whether each material, system or method listed is relevant to your study. If you are not sure if a list item applies to your research, read the appropriate section before selecting a response.

## Materials & experimental systems

| n/a | Involved in the study |
|---|---|
| ☒ | ☐ Antibodies |
| ☒ | ☐ Eukaryotic cell lines |
| ☒ | ☐ Palaeontology and archaeology |
| ☒ | ☐ Animals and other organisms |
| ☒ | ☐ Clinical data |
| ☒ | ☐ Dual use research of concern |

## Methods

| n/a | Involved in the study |
|---|---|
| ☒ | ☐ ChIP-seq |
| ☒ | ☐ Flow cytometry |
| ☒ | ☐ MRI-based neuroimaging |
